# Proteomic Analysis of the Ubiquitin Landscape in the *Drosophila* Embryonic Nervous System and the Adult Photoreceptor Cells

**DOI:** 10.1371/journal.pone.0139083

**Published:** 2015-10-13

**Authors:** Juanma Ramirez, Aitor Martinez, Benoit Lectez, So Young Lee, Maribel Franco, Rosa Barrio, Gunnar Dittmar, Ugo Mayor

**Affiliations:** 1 Department of Biochemistry and Molecular Biology, University of the Basque Country (UPV/EHU), Leioa, Bizkaia, Spain; 2 Functional Genomics Unit, CIC bioGUNE, Derio, Spain; 3 Department of Cellular and Molecular Physiology, Institute of Translational Medicine, University of Liverpool, Liverpool, United Kingdom; 4 Mollecular Cell Biology, Turku Centre for Biotechnology, Turku, Finland; 5 Developmental Neurobiology, Institute of Neurosciences, CSIC/UMH, Sant Joan d’Alacant, Alicante, Spain; 6 Max Delbrück Center for Molecular Medicine, Berlin, Germany; 7 Ikerbasque, Basque Foundation for Science, Bilbao, Bizkaia, Spain; Columbia University, UNITED STATES

## Abstract

**Background:**

Ubiquitination is known to regulate physiological neuronal functions as well as to be involved in a number of neuronal diseases. Several ubiquitin proteomic approaches have been developed during the last decade but, as they have been mostly applied to non-neuronal cell culture, very little is yet known about neuronal ubiquitination pathways *in vivo*.

**Methodology/Principal Findings:**

Using an *in vivo* biotinylation strategy we have isolated and identified the ubiquitinated proteome in neurons both for the developing embryonic brain and for the adult eye of *Drosophila melanogaster*. Bioinformatic comparison of both datasets indicates a significant difference on the ubiquitin substrates, which logically correlates with the processes that are most active at each of the developmental stages. Detection within the isolated material of two ubiquitin E3 ligases, Parkin and Ube3a, indicates their ubiquitinating activity on the studied tissues. Further identification of the proteins that do accumulate upon interference with the proteasomal degradative pathway provides an indication of the proteins that are targeted for clearance in neurons. Last, we report the proof-of-principle validation of two lysine residues required for nSyb ubiquitination.

**Conclusions/Significance:**

These data cast light on the differential and common ubiquitination pathways between the embryonic and adult neurons, and hence will contribute to the understanding of the mechanisms by which neuronal function is regulated. The *in vivo* biotinylation methodology described here complements other approaches for ubiquitome study and offers unique advantages, and is poised to provide further insight into disease mechanisms related to the ubiquitin proteasome system.

## Introduction

Ubiquitination, a process conserved among eukaryotes and present in all type of cells, is a covalent post-translational modification in which an ubiquitin molecule is typically attached to a lysine residue of a protein. This process, carried out by ubiquitin activating (E1), conjugating (E2) and ligating (E3) enzymes, is best known because of its role in protein degradation [1]. However, the versatility of this system to modify proteins with either one or multiple ubiquitins (monoubiquitination or multiubiquitination), even forming ubiquitin chains of different topologies, provides a high complexity capable of regulating other processes such as protein interactions, protein activity or protein localization [2]. Indeed, ubiquitination is now considered to be a key regulator of a wide range of biological processes including those related with the nervous system [3].

The correct performance of the ubiquitin machinery is essential for the proper establishment of neuronal networks, since mutations in different components of the pathway affect the length and number of axons, dendrites and dendritic spines [4]. Other processes, such as neurotransmitter release [5] or neurotransmitter receptor internalization [6], have also been found to be regulated by ubiquitination. It is not surprising then that failures in the Ubiquitin Proteasome System (UPS) are linked to the development of a number of neurological disorders, such as Parkinson’s disease [7], Angelman syndrome [8], and spinal muscular atrophy [9] to cited some [10, 11]. The large-scale identification of the proteins that are being ubiquitinated in the nervous system might therefore provide a starting point for a better understanding of the role of ubiquitination in the brain. Due to the low levels of ubiquitin modified proteins present in cells, together with the rapid speed at which some of them are degraded by the proteasome and the activity of the deubiquitinating enzymes (DUBs), the study of this post-translational modification is however quite a challenge [12].

In recent years, mass spectrometry (MS)-based ubiquitin proteomics have proven to be a good strategy for the identification of ubiquitin-modified proteins from cells. This approach requires a previous enrichment of the ubiquitinated material [13], which has been generally achieved using ubiquitin specific antibodies [14–16], ubiquitin-binding domains (UBDs) [17, 18] or epitope-tagged versions of ubiquitin [19–21]. Some of these methods (i.e. ubiquitin antibodies, UBDs and the hemagglutinin (HA)-tagged version of ubiquitin) require the purification to be performed under native conditions. This usually results in the co-purification of other non-ubiquitinated proteins, and hence can lead to the identification of false positives (i.e. associated proteins that are not actually ubiquitin-conjugated). Recently, a strategy based on antibody purification has successfully detected 1786 ubiquitination sites in 921 proteins from rat brain [22]. The strategy took advantage of the ubiquitin di-glycine-(di-gly)-remnant left in peptides after trypsin digestion, which can be recognized by di-glycine specific antibodies allowing their purification [16]. Nevertheless, this di-glycine signature is common for ubiquitin as well as for other ubiquitin-like proteins, such as Nedd8 or ISG15, which cannot be distinguished by mass spectrometry. Moreover, the di-glycine strategy requires the proteins to be trypsin digested, preventing any immunoblotting on the purified material to validate and further characterize their ubiquitination.

An alternative approach is to carry out the purification under denaturing conditions, in order to remove interacting proteins as well as to protect the ubiquitinated material from the activity of DUBs. Usually polyhistidine tagging has been used for that purpose [19, 23–25]. However, as observed when adequate controls are performed, nickel columns also trap proteins with endogenous histidine-rich motifs, which are very frequent in higher organisms, therefore increasing the identification of false positives.

We recently developed a strategy for efficient isolation of ubiquitin conjugates from flies [21], mice [26] and human cell lines [27]. The system relies on the *in vivo* expression of the biotin ligase (BirA) enzyme from *E*. *coli* as a fusion protein with multiple copies of ubiquitin that bear a short biotinylatable motif at their N-terminus. Endogenous DUBs process this polypeptide precursor into individual ubiquitin molecules that are then biotinylated by BirA *in vivo*. The strength and the specificity of the avidin-biotin interaction allows isolation and enrichment of ubiquitinated proteins, which can then be identified by mass spectrometry. The identified proteins can also be confirmed by Western blotting on the same purified material, further discerning whether they are mono- or poly-ubiquitinated.

Neuronal function and activity are highly context dependent. For instance, during embryonic stages, growth cone extension is required in order for the axons to find their final targets. A number of proteins are involved during this process which will no longer be required once the synaptic target has been recognized [28, 29]. On the other hand, synaptic plasticity of the mature neuron will require extensive remodelling of the cytoskeleton and reallocation of the membranes [30, 31]. Proteins present during the embryonic development of the brain [32] will be regulated differently from those factors necessary for synaptic transmission once the innervations have been established [33], and we do expect ubiquitination to play a significant role for their regulation. In order to compare the processes that are commonly and differentially targeted by ubiquitination during development and later, in mature cells, we present here liquid chromatography-tandem mass spectrometry (LC-MS/MS) data identifying ubiquitinated proteins from *Drosophila* embryonic and adult neurons.

## Materials and Methods

### Fly stocks

The generation of the ^UAS^BirA and ^UAS^(^bio^Ub)_6_-BirA flies and their recombination with elav^GAL4^ for the studying of ubiquitinated material in the embryo nervous system development had been previously described [21]. For the analysis of the ubiquitinated material in *Drosophila* adult brain ^UAS^BirA and the ^UAS^(^bio^Ub)_6_-BirA flies were independently recombined with the eye specific Glass Multimer Reporter-GAL4 driver (GMR^GAL4^). Flies carrying the GMR^GAL4^ (BL 1104), the pan-neuronal elav-GAL4 (elav^GAL4^; BL 8765) or the heat shock-GAL4 (Hs^GAL4^; BL 2077) drivers on the second chromosome and flies carrying the tubulin-GAL4 (Tub^GAL4^; BL 5138) and GAL80 (Tub^GAL80ts^; BL 7017) drivers on the third chromosome were provided by the Bloomington Stock Center (Bloomington, IN, USA). Flies with the glutamatergic neuron-specific GAL4 driver (OK371^GAL4^) on the second chromosome were kindly provided by Cahir O’Kane. Ube3a gain of function and loss of function mutants were a gift from Janice Fischer [34]. Flies overexpressing the C-terminal half of Rpn10 (^UAS^Rpn10-ΔNTH) were a gift from Zoltan Lipinszki [35].

### Fly sample material collection

Embryo sample collection was performed by enclosing flies, at 25°C, in fly cages with Petri dishes containing an apple juice-rich agar layer (2.25% agar, 2.5% dextrose, 25% apple juice and 0.25% Nipagin in distilled H_2_O), partially covered with yeast paste as previously described [21]. For adult sample collection flies were grown, unless otherwise specified, at 25°C in wheat flour and yeast medium (1% agar, 5.5% dextrose, 3.5% wheat flour, 5% yeast, 0.25% Nipagin, 0.4% propionic acid and 0.02% benzalkonium chloride in distilled H_2_O). Adult flies 2–5 days old were flash frozen in liquid nitrogen. Heads were severed by shaking the frozen flies and separated from the remaining body parts by using a pair of sieves with a nominal cut-off of 710 and 425 μm.

### Extract preparation and biotin based pulldown

About 1 g of whole dechorionated embryos (stages 13–17) or ~0.35 g of heads of 2–5 days old flies were homogenized under denaturing condition in 2.9 mL Lysis buffer (8M Urea, 1% SDS and 50 mM N-ethylmaleimide in PBS, including a protease inhibitor cocktail from Roche Applied Science). The biotin pulldown was performed on the homogenates as previously described [13, 21, 26]. Briefly, embryo and adult lysates were incubated with 200 ΔL and 300 ΔL of NeutrAvidin-agarose beads (Thermo Scientific) suspension for approximately 3 h, respectively. Afterwards, beads were subjected to stringent washes with the following washing buffers (WB): 8 M Urea and 1% SDS (WB1), 6 M Guanidine-HCl (WB2), 6.4 M Urea, 1 M NaCl and 0.2% SDS (WB3), 4 M Urea, 1 M NaCl, 10% Isopropanol, 10% Ethanol and 0.2% SDS (WB4), 8 M Urea and 1% SDS (WB5) and 2% SDS (WB6). All buffers were prepared in PBS. Elution of beads-bounded material was performed by heating beads at 95°C in the Elution Buffer (4X Laemmli Buffer and 100 mM of DTT). The Elution Buffer volume was half of the NeutrAvidin-agarose beads suspension used (i.e. 100 ΔL and 150 ΔL). The recovered volumes were ~130 ΔL for embryo samples and ~150 ΔL for adult samples. In both cases the isolated biotinylated proteins boiled off the beads gave a typical recovery yield of 20–40%.

### Western blotting

The following antibodies were used: goat anti-biotin-horseradish peroxidase (HRP) conjugated antibody (Cell Signalling) at 1:200 for embryo samples and 1:1000 for adult samples; chicken polyclonal anti-BirA antibody (SIGMA) at 1:1000; mouse monoclonal anti-GFP antibody (Roche Applied Science) at 1:1000; mouse monoclonal anti-Flag M2-HRP conjugated antibody (Sigma) at 1:1000; mouse monoclonal anti-Syx1A antibody (DSHB) at 1:50; rabbit polyclonal anti-Eps15 antibody [36] at 1:150; rabbit polyclonal anti-Fax antibody (a gift from Eric Liebl) at 1:1000; mouse monoclonal anti-Atpα antibody (DSHB) at 1:50; rabbit polyclonal anti-Parkin antibody (a gift from Alex Whitworth) at 1:3000; rabbit polyclonal anti-TBPH antibody (a gift from Raffaella Klima) [37] at 1:50; rabbit polyclonal anti-Ube3a antibody (gift from Fen-Biao Gao) [38] at 1:1000; rabbit polyclonal anti-Tan antibody [39], kindly provided by Professor Bernhard Hovemann, at 1:100; mouse monoclonal anti-Rpn10 antibody (gift from Zoltan Lipinszki) at 1:100 [35]; mouse monoclonal anti-FK1 antibody (Enzo Life Sciences) at 1:1000; mouse monoclonal anti-Nrt (DSHB) at 1:20; guinea pig polyclonal anti-Lqf [40] at 1:200 and HRP labelled secondary antibodies from Jackson ImmuoResearch Laboratories. Depending on the antibody, between 0.001% and 0.2% of the input samples and 5–10% of the elution samples were loaded. Generally, 4–15% gradient gels (bioRAD) were used. Western blotting and transfer to PVDF membranes were performed using the iBlot system (Invitrogen). The membranes were developed using ECL kit (GE Healthcare) following the manufacturer’s instructions. Dual-colour Western blots were prepared by assigning independent colour channels to two independent Western blots developed in the same membrane.

### Silver staining

About 10% of the elution samples were run in 4–15% gradient gels (Biorad). Afterwards, gels were fixed in 40% ethanol and 10% acetic acid containing solution. After fixation, silver staining was performed with a SilverQuest kit (Invitrogen) following the manufacturer's instructions.

### Mass spectrometry

The identification of ubiquitinated material was performed from four or three independent pull-down experiments for embryo or adult, respectively. For the analysis of the proteins accumulating when C-terminal half of Rpn10 is expressed three independent biological replicates were done for each embryos and adults. The MS analysis was carried out as in Lectez *et al*. (2014) [26]. Basically, eluted samples were run on a SDS-PAGE for approximately 4 mm into the separating gel, which was afterwards cut in four slices. Proteins were converted to peptides using an automated in-gel digestion protocol [41, 42], and then separated on a 15 cm reverse phase column (packed in house, with 3 Δm Reprosil beads, Dr. Maisch GmbH) using a 5 to 50% acetonitrile gradient (Proxeon nano nLC). Peptides ionization was performed on a proxeon ion source and sprayed directly into the mass spectrometer (Q-Exactive or Velos-Orbitrap, Thermo Scientific). The MaxQuant software package (version 1.3.0.5), with top 10 MS/MS peaks per 100 Da and 1% FDR for both peptides and proteins [43], was used for the analysis of recorded spectra. Searches were performed using the Andromeda search engine against the Uniprot *Drosophila melanogaster* database. As a fixed modification, cysteine carbamidomethylation was selected and as variable modifications, methionine oxidation, protein N-terminal acetylation and di-glycine addition on the ε-aminogroup of lysines. Two missed trypsin (full specificity) cleavages were allowed. Mass tolerance of precursor ions was set to 6 ppm and for fragment ions to 20 ppm. The identified ubiquitylation sites were checked using in house programmed software tools. Label Free Quantification was also performed with MaxQuant.

### Cloning and point mutations

N-terminal and C-terminal GFP-tag, as well as Flag-tagged ubiquitin were cloned and inserted into pAc5 (Invitrogen) vectors as previously described [44]. The fly *nSyb* gene was amplified from a *Drosophila* cDNA library using the *nSyb-Fw* (GCCCTCGAGATGGCGGACGCTGCACCAGCTGG) and *nSyb*-*Rv* (GCCGCTAGCCACGCCGCCGTGATCGCC) primers and inserted into the N-GFP or C-GFP pAc5 vectors between the *XhoI* and *NheI* enzyme sites, for carrying either an N-terminally (pAc5.1-NGFP-nSyb) or C-terminally (pAc5.1-nSyb-CGFP) GFP tag. nSyb lysine to arginine mutants carrying either the lysine 71 or the lysine 78 mutated to arginine (K71R and K78R) and a double mutant carrying both (DM: K71R/K78R) were generated on the pAc5-nSyb-CGFP vector using the QuikChange Site-Directed Mutagenesis Kit (Stratagene) according to manufacturer’s instructions. The primers used for mutagenesis were the following:

*nSyb-K71R-Fw* (CGCACGAACGTGGAGAGGGTGCTGGAGCGCGAC)
*nSyb-K71R-Rv* (GTCGCGCTCCAGCACCCTCTCCACGTTCGTGCG)
*nSyb-K78R-Fw* (CTGGAGCGCGACAGCAGGCTGTCGGAGCTGGACG)
*nSyb-K78R-Rv* (CGTCCAGCTCCGACAGCCTGCTGTCGCGCTCCAG).

### BG2 cell culture, transfection and GFP pulldown

*Drosophila* BG2 neuronal cells [45] were kindly provided by the Drosophila RNAi Screening Center (DRSC). They were cultured in Shields and Sang M3 Insect Medium (Sigma) supplemented with 10% FBS (Invitrogen), 1:100 penicillin/streptomycin, 20 μg/mL of insulin (Sigma) and 50% conditioned medium (medium removed from cultured cells and centrifuged 5 min at 2500 rpm) at 25°C. Calcium phosphate transfection method was used to transfect 2 Δg of the *nSyb* and 0.4 Δg of the Flag-tagged *ubiquitin* genes into 3 x 10^6^ cells. After 18 h, cells were washed with 1X PBS and new supplemented M3 medium was added. Cells were then further incubated for additional 48 h, after which they were subjected to GFP pulldown using GFP beads (Chromotek GmbH) as previously described [44].

### Bioinformatic and statistical analysis

The proteins identified by MS were analysed for functional interpretation with GO Term Mapper (http://go.princeton.edu/cgi-bin/GOTermMapper), a tool for the classification of genes into high-levels GO parent terms (GO Slim) and with g:Cocoa, a tool integrated in the g:Profiler web server to perform comparative analysis of multiple gene list [46]. A t-test was applied to every pair of nSyb variants for statistical analysis. Normal distribution was assessed by the Kolmogorov-Smirnov test and equality of variance with F test. All statistics were performed with GraphPad.

## Results and Discussion

### Expansion of the bioUb strategy from embryonic to adult neurons

**Fig 1:**
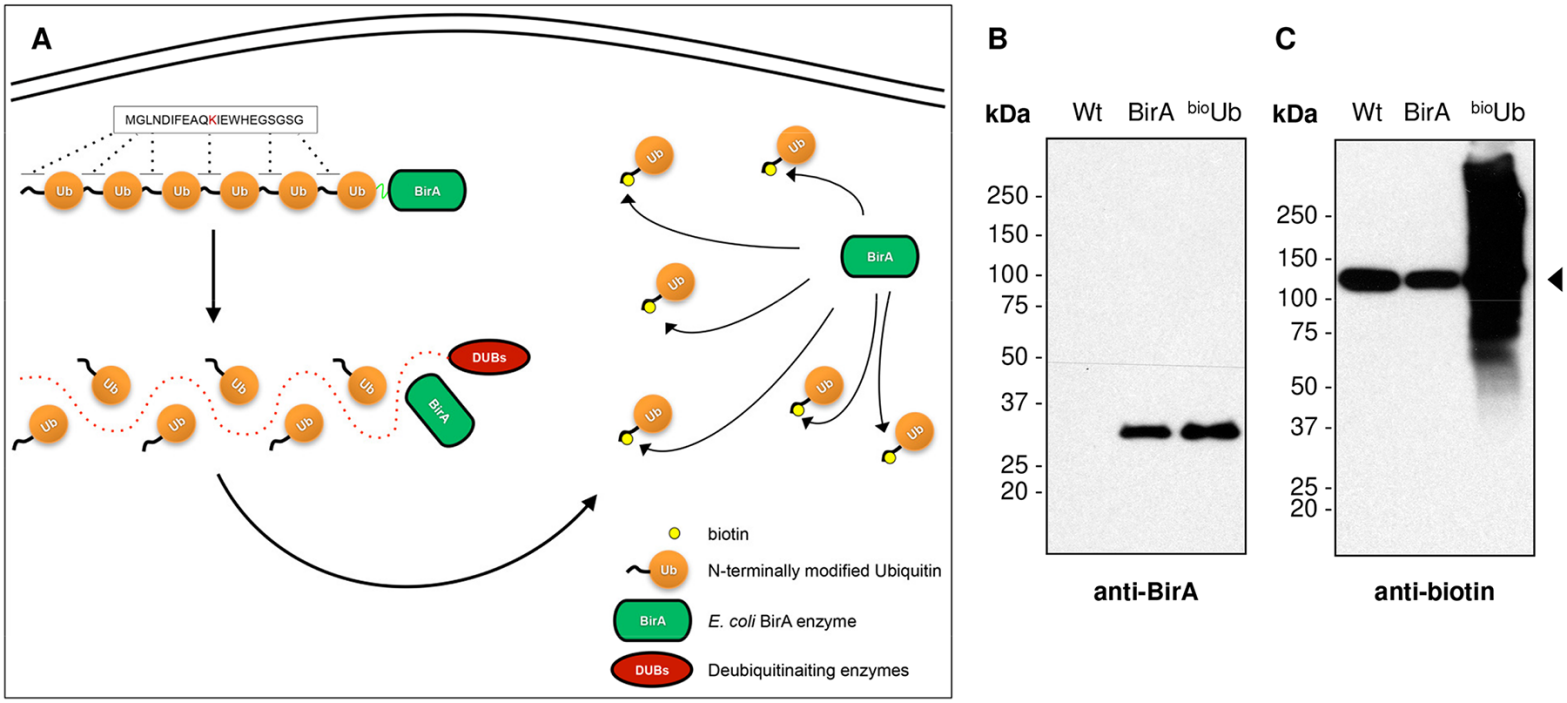
Biotinylated ubiquitin is incorporated into conjugates in the *Drosophila* adult eye. (A) Schematic illustration of the *in vivo* biotinylation of ubiquitin [21]. The construct is expressed as a polyubiquitin chain fused to the *E*.*coli* BirA enzyme, which is digested by the endogenous deubiquitinating enzymes (DUBs). While this clearly indicates that DUB enzymes are capable of deconstructing linear ^bio^Ub chains, we have no evidence to support that E2/E3 enzymes are actually capable of constructing those linear ^bio^Ub chains the same way they would do with the more abundant endogenous ubiquitin. Afterwards, the BirA enzyme recognizes the short motif incorporated at the N-terminus of each ubiquitin (sequence is indicated) and attaches a biotin molecule to its lysine residue (red). The biotinylated ubiquitins are then conjugated to the target proteins. (B) Western blot with anti-BirA antibody using total *Drosophila* head extracts confirmed the full digestion of the ^bio^Ub precursor by endogenous DUBs. No undigested forms of the precursor were found above the expected molecular size of BirA (35 kDa). (C) Anti-biotin Western blot on the same total extracts confirmed the biotinylation and conjugation of the GMR^GAL4^–driven expressed ubiquitin. An endogenous protein known to be biotinylated (CG1516) appeared in all the samples (arrowhead). The expected smear corresponding to biotinylated ubiquitin conjugates was only present in the ^bio^Ub sample. WT: *Oregon R*; BirA: *GMR* ^*GAL4*^ */CyO*;^*UAS*^ *BirA/TM6*; ^bio^Ub: *GMR* ^*GAL4*^,^*UAS*^ *(* ^*bio*^ *Ub)*_*6*_ *-BirA/CyO*.

The first application of the ^bio^Ub strategy (Fig 1A) was directed to identifying the ubiquitinated proteome during nervous system development. For this purpose we used elav^GAL4^ flies expressing the ^UAS^(^bio^Ub)_6_-BirA (^bio^Ub) and ^UAS^BirA (BirA) constructs in the nervous system of *Drosophila melanogaster* embryos [21]. In order to elucidate whether ubiquitination regulates different processes in the adult nervous system, we initially tested expression levels with elav^GAL4^ flies in the adult fly heads. This promoter, however, does not reach the expression required for proteomic studies (see S1 Fig), so in order to apply our strategy to adult neurons, we overexpressed the same constructs under the control of the eye specific Glass Multimer Reporter-GAL4 driver (GMR^GAL4^). This driver is also expressed on a number of different cell types [47], but has been found to be an ideal system to model and investigate neurodegenerative disorders [48]. More importantly, it provided the best protein expression level of our construct, as required for proteomic studies, in comparison with other drivers tested (S1 Fig). Western blots from whole head extracts with an antibody that recognizes the *E*. *coli* BirA enzyme showed that the ^UAS^(^bio^Ub)_6_-BirA precursor was fully digested by endogenous deubiquitinating enzymes (DUBs) when expressed under the control of GMR^GAL4^. Only one band, corresponding to the BirA enzyme, was observed in both the BirA and ^bio^Ub samples (Fig 1B); the full-length precursor was not detected. Based on anti-biotin immunoblotting, expression of only the BirA enzyme by GMR^GAL4^ did not cause promiscuous biotinylation of endogenous proteins (Fig 1C). However, in the ^bio^Ub sample, high molecular weight proteins were detected with the anti-biotin antibody, which correspond to proteins conjugated with biotinylated ubiquitin.

### Identification by Mass Spectrometry of the neuronal ubiquitin landscape in embryonic and adults samples

**Fig 2:**
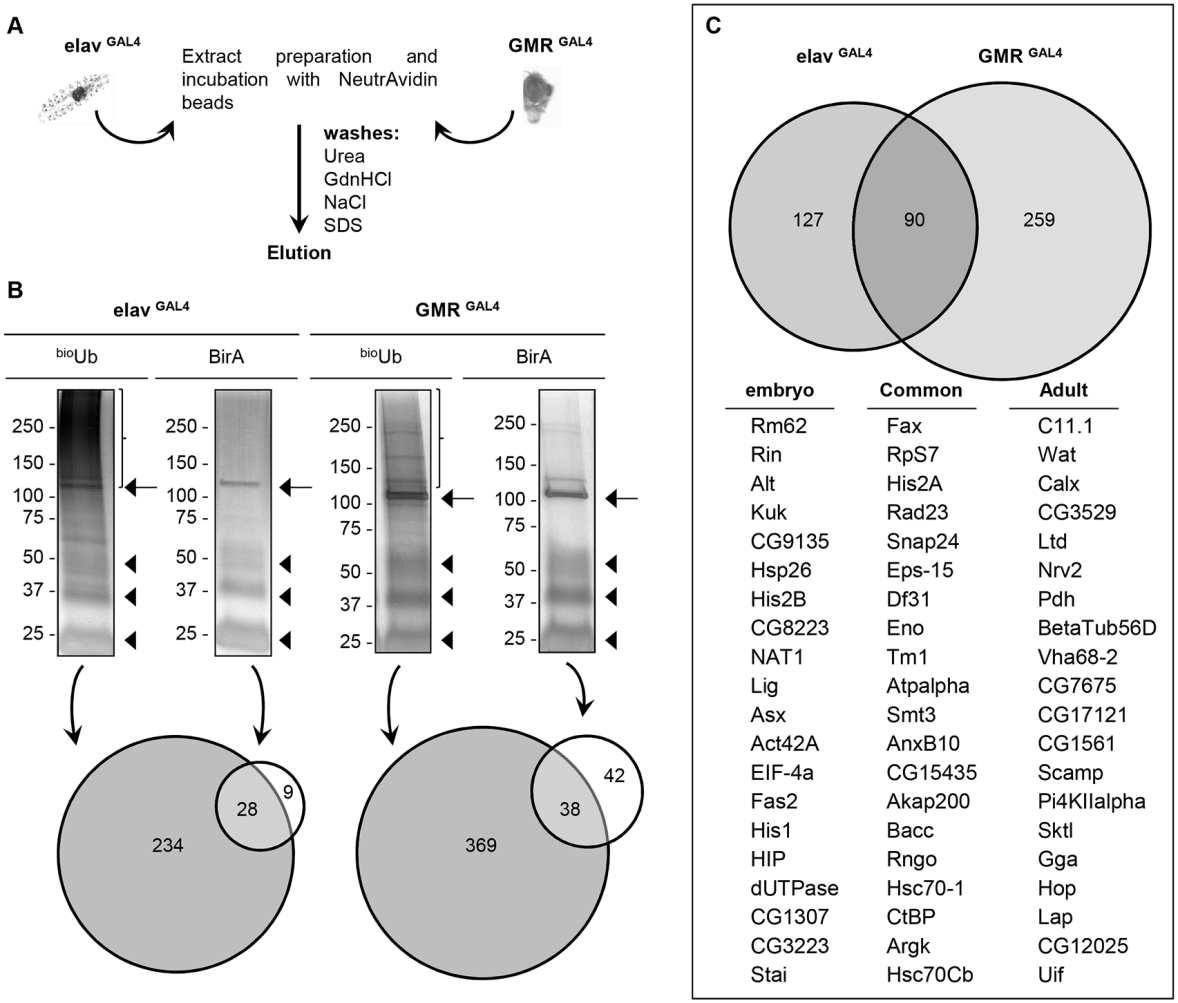
Strategy for pulldown of ubiquitinated material from *Drosophila melanogaster* tissues. (A) Schematic illustration of the strategy applied to purify ubiquitinated material from *Drosophila* embryo nervous system and adult eye. Both embryo and adult samples, expressing either the ^UAS^(^bio^Ub)_6_-BirA or the ^UAS^BirA (control) constructs, were homogenized, clarified and incubated with High Capacity NeutrAvidin agarose resin. Beads were then subjected to stringent washes to remove the non-biotinylated proteins. Afterwards, material bound to beads was eluted by applying a heat treatment. (B) Silver staining of the eluted material revealed no protein in the BirA samples except for the endogenously biotinylated proteins, particularly the most abundant (CG1516) (arrows), while in the ^bio^Ub samples the typical smear of ubiquitinated material was detected (brackets). Monomer, dimer and tetramer forms of NeutrAvidin molecules leaking from the beads were also found in all the samples (arrowheads). Mass spectrometry analysis performed with embryo samples identified 37 and 234 proteins in the control and experimental samples, respectively. In adult the analysis identified 80 proteins in the control and 369 in the experimental samples. (C) Venn diagram showing the distribution of the identified ubiquitin conjugates. Identified ubiquitin carriers are listed in Table 1. Only 90 ubiquitin conjugates were found to be present in both data sets. The top 20 ubiquitin conjugates found only in embryo, only in adult and those found in both samples are listed below the Venn diagram. All peptides and intensities of the different analysis are shown in S1 and S2 Tables.

In order to compare the ubiquitin landscape in embryonic and adult nervous systems, both BirA and ^bio^Ub extracts from *Drosophila* embryos of stage 13–17 and from 2–5 days old adult heads were prepared under denaturing conditions and incubated with NeutrAvidin agarose beads to capture the biotinylated material (Fig 2A). The purified material was analysed by silver staining (Fig 2B, S2 Fig) and immunoblotting (S3 Fig), to confirm that the purifications worked successfully. Four biologically independent pull-down experiments were performed from embryo and three from adult fly heads, with their corresponding BirA controls, and independently subjected to LC-MS/MS analysis. The treatment to which biological samples are subjected for MS analysis usually introduces, either deliberately or unintentionally, foreign artefacts or contaminants that can potentially interfere with the results. Among them, trypsin enzymes used for digestions, keratins, found in hair, skin and dust, or other protein commonly used in different lab techniques, such as bovine serum albumin, or protein A and G, are the most frequent ones [49]. In our analysis, proteins reported to be common contaminants in MS analysis were excluded from the MS list. They were mostly trypsin, keratin and albumin precursors. Identified proteins corresponding to other species different to *Drosophila* were also excluded. After subtraction of these contaminants and background proteins a total of 234 proteins were identified as ubiquitinated material in the *Drosophila* nervous system during stage 13 to stage 17 of the embryo development (Fig 2B, S1 Table). All but 25 of previously identified proteins [21] were confirmed in this new analysis. In the case of the adult brain a total of 369 proteins were identified (Fig 2B, S2 Table). However, not all the proteins identified were found in every biological replicate (S4 Fig). Indeed, about 67% of all the proteins identified in elav^GAL4^ samples, and 56% in GMR^GAL4^, were found only in one biological replica. This variability, however, positively correlates with the maximum Label-Free Quantification (LFQ) intensity recorded for each protein among the different replicas, so those proteins appearing in more independent experiments are the ones with higher LFQ Intensity values, and vice versa (S5 Fig). LFQ is used for quantifying the levels of proteins identified by MS without the need of using protein isotopic labelling [50]. In our MS data, about 80% (embryo) and 68% (adult) of the proteins identified just in one biological replica showed a maximum LFQ intensity record lower than 1x10^6^, suggesting that the group of proteins identified only in one experiment are the less abundant ones. The levels of ubiquitinated proteins found under physiological conditions are usually very low, because some of them are rapidly degraded upon ubiquitination [51], and/or are only ubiquitinated in well-defined temporal windows [52] and/or are subjected to the activity of deubiquitinating enzymes [53]. It is therefore not surprising that some proteins appear in one proteomic experiment but not in others, since low abundant proteins might sometimes escape from being either captured in the purification steps or from the detection by the mass spectrometer.

**Fig 3:**
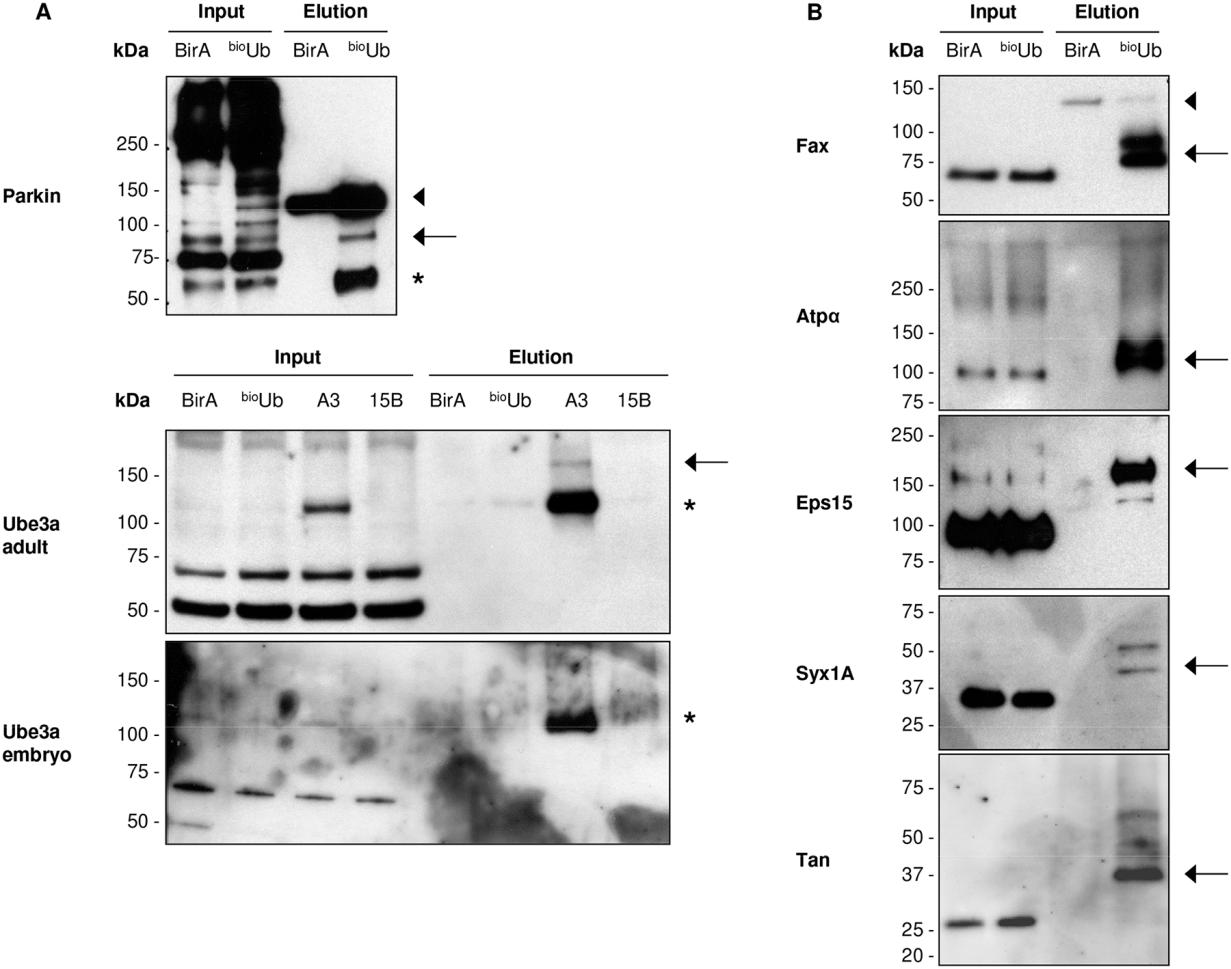
Western blot validation of identified ubiquitin conjugates and ubiqutin carriers. (A) Western blot performed with antibodies against Parkin (upper panel) or Ube3a E3 ligases (bottom panels). The ability of the HECT-type, as well as RING between RING-type E3 ligases to form thioester linkages with the ubiquitin before they transfer it to the substrates allow us to trap them while they are carrying ubiquitin. Since the reducing agents used to perform the elution from the beads breaks this type of linkage, there is no increase in their molecular weight relative to the inputs (*). A fraction of both E3s appears also conjugated with ubiquitin despite the DTT (arrows). This is probably due to auto-ubiquitination at some lysine residue. Parkin antibody non-specifically recognized several proteins in the inputs. The appropriate Parkin band is the one at 55 kDa. (B) Western blots with specific antibodies to some of the proteins identified in the adult pulldown revealed the expected increase of their molecular weight in the ^bio^Ub sample relative to the inputs. Covalent attachment of ubiquitin should increase the protein’s molecular weight by about 10 kDa for each ubiquitin attached. Therefore, the increase shown in the Western blots (arrows) reflects their ubiquitinated status. Endogenous biotinylated CG1516 protein, non-specifically identified by some antibodies, is marked with an arrowhead. All Western blots were performed with adult samples, except Ube3a that was also test in embryo. BirA: *GMR* ^*GAL4*^ */CyO*;^*UAS*^ *BirA/TM6*; ^bio^Ub: *GMR* ^*GAL4*^,^*UAS*^ *(* ^*bio*^ *Ub)*_*6*_ *-BirA/CyO*; A3 (Ube3a overexpression): *GMR* ^*GAL4*^,^*UAS*^ *(* ^*bio*^ *Ub)*_*6*_ *-BirA/CyO*;^*UAS*^ *Dube3A/TM6B*; 15B (Ube3a deletion mutant): *GMR* ^*GAL4*^,^*UAS*^ *(* ^*bio*^ *Ub)*_*6*_ *-BirA/CyO*, *Dube3A* ^*15B*^ */TM6B*.

Comparison of the ubiquitin proteome between the adult dataset (369 proteins) and the embryonic dataset (234 proteins) resulted in only 103 proteins being found to be common between them. Of these, 13 were known ubiquitin-conjugating enzymes and the remaining 90 were categorized as ubiquitin conjugates (Fig 2C). Nevertheless, the fact that only ~20% of the total proteins identified are common does not discard the possibility of other proteins being ubiquitinated in both tissues as well but being missed due to their low levels. In fact, we were able to detect the ubiquitination of Syntaxin 1A (Syx1A) during embryo development (Fig 3B) [21], despite this protein not being detected in neither of the MS analysis performed with embryo samples so far. Similarly, we also observed the ubiquitination of the TAR DNA-binding protein-43 homolog (TBPH) in the adult eye (S3 Fig), despite this protein not being identified in the MS analysis. The ubiquitinated fraction of some proteins might remain undetected in living tissues due to their low levels; however, the differences observed here between the ubiquitin proteomes of developing and mature neurons, in this case differentiated photoreceptor cells, are high enough to suggest that certain proteins are preferentially ubiquitinated in specific cell types during specific periods of the *Drosophila* life cycle. These data therefore emphasize the importance of using the appropriate cell type when studying ubiquitination.

### Active ubiquitin conjugating enzymes are identified in both developing and adult neurons

In a previous study, 11 active ubiquitin carriers (i.e. proteins that are not conjugated with ubiquitin, but transporting it) were identified among the proteins isolated from the embryonic nervous system [21]. Active E2 conjugating enzymes and some E3 ubiquitin ligases (those classified as HECT, and also the RING between RING E3 ligases) generate a thioester linkage in its active-site cysteine with ubiquitin before transferring it to substrates [54, 55]. This thioester linkage is kept intact through our pulldown until the elution step, when it is broken by the addition of DTT [21, 26, 27], allowing us to isolate those enzymes that are carrying ubiquitin. Here, a total of 24 ubiquitin carriers, listed in Table 1, were identified by MS. Some of the E2s were only identified in embryos (CG5823, CG8188) or adults (TGS101, Ubc6), while others appeared in both tissues (CG2924, Morgue, Ubc10). In addition to Ube3a, which was already identified in our earlier work, we now identified six more HECT type E3 ligases (Ctrip, CG5604, CG5087, Nedd4, Park and Su(dx)), most of them in the adult sample (Table 1). In the past, we validated the carrier status of E1 and E2 enzymes [21], but here we decided to validate the carrier status of some E3 ligases.

**Table 1:** Ubiquitin carriers identified in the ^bio^Ub pulldown.

				elav		GMR	
CG number a	Protein description a	Gene symbol a	Mass	PEP Score	n	PEP Score	n
**Ubiquitin activating enzyme**							
CG1782	Ubiquitin activating enzyme 1	Uba1	130,8	0	4	0	3
**Ubiquitin conjugating enzymes**							
CG40045	-	CG40045	19,4	0	4	0	2
CG5788	Ubiquitin conjugating enzyme 10	Ubc10	17,9	1.35 x 10^−087^	4	1.09 x 10^−142^	1
CG4443	Courtless	crl	18,5	2.94 x 10^−032^	4	2.60 x 10^−035^	2
CG18319	Bendless	ben	17,2	4.16 x 10^−195^	4	2.33 x 10^−205^	1
CG7656	-	CG7656	34,7	1.04 x 10^−086^	3	9.79 x 10^−274^	3
CG2924	-	CG2924	44,4	2.12 x 10^−026^	3	7.91 x 10^−029^	2
CG2257	Ubc-E2H	Ubc-E2H	20,9	4.26 x 10^−136^	3	1.32 x 10^−075^	1
CG7425	Effete	eff	16,7	1.05 x 10^−009^	2		
CG8284	Ubiquitin conjugating enzyme 4	Ubc4	22,5	2.89 x 10^−142^	2	5.18 x 10^−003^	1
CG6720	Ubiquitin conjugating enzyme 2	UbcD2	24,4	9.87 x 10^−048^	2	2.59 x 10^−007^	1
CG5823	-	CG5823	30,7	1.08 x 10^−003^	1		
CG8188	-	CG8188	23,3	1.15 x 10^−006^	1		
CG15437	Modifier of rpr and grim, ubiquitously expressed	morgue	55,9	1.47 x 10^−010^	1	3.66 x 10^−020^	2
CG10682	Vihar	vih	19,8	2.05 x 10^−040^	1		
CG9712	Tumor susceptibility gene 101	TSG101	45,2			3.53 x 10^−091^	1
CG2013	Ubiquitin conjugating enzyme 6	Ubc6	13,4			1.25 x 10^−026^	1
**Ubiquitin ligase enzymes**							
CG6190	Ubiquitin protein ligase E3A	Ube3a	107,6	5.07 x 10^−019^	2	8.63 x 10^−016^	2
CG42574	Circadian trip	ctrip	336,6	5.39 x 10^−004^	1	9.35 x 10^−003^	1
CG42279	Nedd4	Nedd4	113,4			5.94 x 10^−142^	3
CG5604	-	CG5604	302,2			2.95 x 10^−019^	2
CG4244	Suppressor of deltex	Su(dx)	108,0			9.19 x 10^−021^	2
CG5087	-	CG5087	123,8			5.70 x 10^−003^	2
CG10523	Parkin	park	54,1			7.61 x 10^−013^	1

Posterior Error Probabilities (PEP Score) and number of identifications in independent ^bio^Ub pulldown experiments (n) are reported. All peptides and intensities are shown in S1 and S2 Tables.

^*a*^ CG number, protein description and gen symbol given according to flybase nomenclature (www.flybase.org).

When a protein is ubiquitinated on a lysine, its purified ubiquitinated fraction should appear at a higher molecular weight due to the additional mass of the conjugated ubiquitin(s). However, ubiquitin carriers should not show any molecular weight increase in the elution fraction, relative to the input fraction. The ubiquitin that is conjugated via a thioester bond to the active-site cysteine of an ubiquitin carrier enzyme will break away when eluting the sample from the beads with a DTT-rich buffer. In this case, the purified carrier enzyme will therefore display the same molecular weight as in the input lane. Based on antibody availability, we were able to validate, using this criteria, Parkin and Ube3a as active ubiquitin carriers. A Western blot using anti-Parkin (Fig 3A) showed a band of the appropriate size (~55 kDa), same size in the input and elution of the ^bio^Ub sample, is consistent with the majority of parkin being pulled down from the *Drosophila* eye cells because is actively carrying ubiquitin via thioester linkage, as opposed to being conjugated to ubiquitin via substrate lysines. In the case of Ube3A, the fraction carrying ubiquitin was close to the limits of detection due to its low level, and was hindered because the antibody cross-reacted with one of the endogenously biotinylated proteins (CG1516). However, when combining the ^bio^Ub flies (both elav^GAL4^ and GMR^GAL4^) with flies overexpressing Ube3a (^UAS^Ube3a), we were able to confirm that overexpressed Ube3a is also an ubiquitin carrier (Fig 3A).

### Differences between the ubiquitinated landscapes in neurons of the developing embryo and the adult photoreceptor cells

Having analysed the ubiquitin carrier enzymes separately, the remaining identifications are expected to correspond to lysine-ubiquitinated proteins, some of which were validated by Western blotting to characterize the number of ubiquitin molecules attached (Fig 3B and Franco *et al*., 2011 [21]). The total numbers of ubiquitin conjugates identified in embryonic and adult samples were, respectively, 217 and 349, with only 90 being common to both data sets (Fig 2C). A short list of the top-20 ubiquitin conjugated proteins, based on the number of independent identification in different replica and in the Posterior Error Probabilities (PEP), is also given for each of the datasets (Fig 2C).

**Fig 4:**
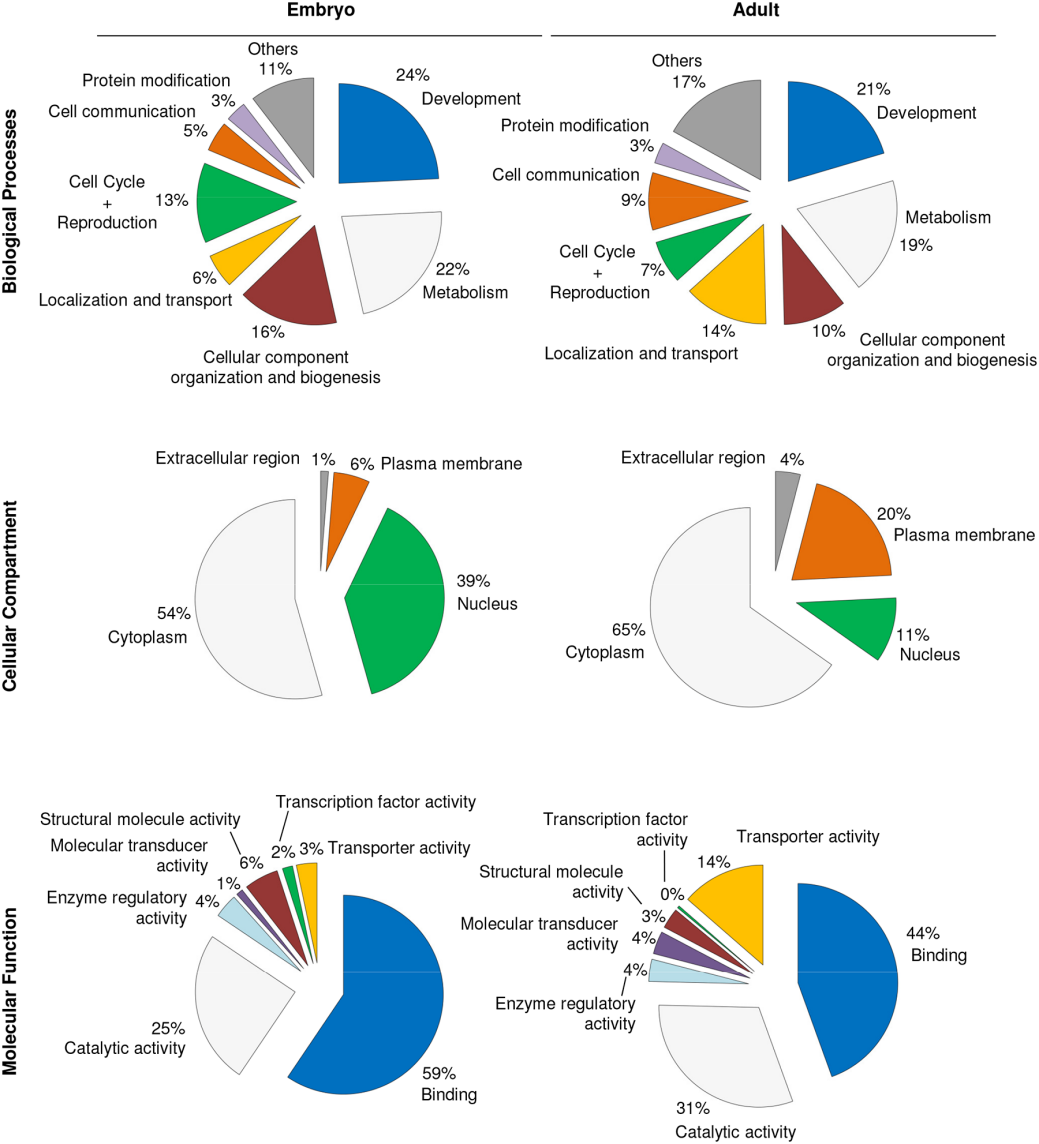
Functional interpretation of the identified ubiquitin conjugates from embryo and adult samples. Ubiquitin conjugates present either in embryo or in adult pulldowns were further analysed with GO Term mapper. The analysis provided a list of broad GO terms (GO Slim) for the Biological Process (72 categories), Cellular Compartment (30 categories) and Molecular Function (44 categories) domains, which were additionally grouped into fewer categories to make their representation more visual and understandable. In the Biological Process pie chart categories representing less than 3% were grouped into “others”. In the Molecular Function pie chart biggest groups are provided. In the Cellular Compartment pie chart only “extracellular matrix”, “plasma membrane”, “nucleus” and “cytoplasm” categories are depicted.

For the purpose of obtaining a functional overview of the main pathways regulated by ubiquitination at those two stages, a bioinformatic analysis was performed with GO Term mapper (http://go.princeton.edu/cgi-bin/GOTermMapper), which provides a list of high-level or broader GO Terms categories (GO Slims) [56], and g-profiler, which performs comparative analysis of multiple gene lists and provides a hierarchically sorted list of enriched GO terms [46]. As illustrated with the pie charts for the gene’s biological processes, localization and activities, significant differences exist between the proteins found only in embryonic or only in adult samples (Fig 4). Most notably, we found a shift in the “Biological processes” domain from a predominance of “Cell cycle + Reproduction” and “Cellular component organization and biogenesis” terms in the embryo to “Localization and transport” and “Cell communication” in the adult samples. Concomitantly, in regards to “Cellular compartment”, we found a shift from a predominance of proteins with nuclear localization in the embryonic neurons to plasma membrane in the adult ones. When analysing the “Molecular function” domain, a significant shift was observed from “Structural molecule” and “Transcription factor” activities in the embryo to the “Transporter activity” and “Molecular transducer activity” categories in the adult. Similarly, the g:profiler analysis (S3 Table) showed the specific enrichment of terms such as “Cell cycle”, “Developmental process” and “Cellular component organization or biogenesis” in the analysis performed with the proteins found only in embryo. These biological processes did not appear enriched in the analysis with the proteins found only in adult, but instead “Localization”, “Synaptic transmission” and “Establishment of localization” did. Differently enriched terms in regards to the “Cellular compartment” and “Molecular function” domains were also found. Terms as “Macromolecular complex”, “Cytoskeleton”, “Nucleus” and “mRNA binding” were enriched in the embryonic sample, while “Plasma membrane”, “Synapse”, “Transporter activity” and “SNAP receptor activity” terms were enriched in the adult sample. In our view, all three GO term domains (i.e.: Biological process, Cellular compartment and Molecular function) display a common pattern. The embryonic proteins that are more abundantly ubiquitinated are those that need to be regulated in order for the neuron to reach a mature stage, either in terms of transcriptional regulation in the nucleus or through the growth and establishment of the axonal structure. Once mature, the proteins involved in signalling and communication between neurons, and therefore located at the cell membrane, are found ubiquitinated in the adult samples.

### Changes in the ubiquitinated landscape upon interference with proteasomal function

Most ubiquitin proteomic studies have been performed on cell cultures on which the proteasome had been inhibited, with the intention to lead to a significant accumulation of proteins that would otherwise be degraded as determined by their ubiquitination status. Our ^bio^Ub strategy, thanks to the high affinity as well as capability to withstand denaturing conditions, does in principle not require blocking of the proteasome in order to detect hundreds of proteins. However, for the purpose of comparison, we decided to investigate the changes that can be caused by blocking the proteasome, in this case by the overexpression of the C-terminal half of the proteasomal shuttling protein Rpn10 (^UAS^Rpn10-ΔNTH; hereinafter Rpn10^DN^) (panel A in S6 Fig). Rpn10^DN^ contains three Ubiquitin Interacting Motifs (UIM) that recognize and bind polyubiquitinated material, but lacks the von Willebrand factor A (VWA) domain required for binding to the proteasome [35]. Overexpression of Rpn10^DN^ has been shown to act in a dominant negative manner by binding to ubiquitinated material and preventing its delivery to the proteasome, resulting in a significant accumulation of proteasome-targeted Rpn10 cargo [35, 44].

Three independent biological replicas, from both embryo and adult, overexpressing Rpn10^DN^ were subjected to NeutrAvidin pulldown and MS analysis as described above. In agreement with previous reports [35, 44], overexpression of Rpn10^DN^ resulted in an increase of high molecular-weight ubiquitinated material isolated with our ^bio^Ub pulldown, as measured by silver staining and Western blot (S6 and S7 Figs). Surprisingly, the MS analysis provided a reduced number of total identifications in both embryonic and adult Rpn10^DN^ experiments (S8 Fig). The pool of ectopic biotinylated ubiquitin is limited; therefore, we hypothesise that the accumulation by Rpn10 of polyubiquitinated proteins will block ubiquitin recycling and will therefore result in a significant depletion of available biotinylated ubiquitin molecules. While the ubiquitin substrates trapped by the C-terminal half of Rpn10 are neither processed by the proteasome probably nor by deubiquitinases, as observed for other ubiquitin binding domains [57], ubiquitination of other proteins will be significantly reduced and therefore their isolation will also be constrained. We indeed detected a reduction for monoubiquitinated proteins, despite their ubiquitination expected to be constant with the proteasome blockade (panel D in S6 Fig). This idea is also supported by anti-biotin immunoblotting, where a stronger signal of higher molecular weight biotinylated proteins is detected when Rpn10^DN^ is expressed in adult (panel C in S6 Fig).

The various datasets in this work were obtained from independent biological samples, and the experiments performed independently, therefore we could not jointly analyse all our data sets with adequate statistical criteria (S1 and S2 Tables). Moreover, comparison of protein abundance between two conditions requires of a proper quantifiable value. Label free quantification (LFQ) has proven to be a proper measurement for that purpose [50], however, in some circumstances, mass spectrometric software does not obtain enough data to provide a LFQ value for a given identified protein, in which cases LFQ value is shown as 0, making difficult the statistical analysis. For that reason, LFQ intensity ratios between pairs of Rpn10 and non-Rpn10 ^bio^Ub samples were determined and used as selection criteria to discriminate among those proteins found more ubiquitinated upon Rpn10^DN^ overexpression for each data pair. Proteins identified in at least two independent Rpn10^DN^ biological replicates and whose Rpn10/^bio^Ub LFQ intensity ratio was bigger than 4 in at least two experiments were preliminary selected as candidate Rpn10 substrates. Among the proteins identified in Rpn10^DN^ embryo samples none of them completely fulfil the applied criteria. However, among the ones identified in adult Rpn10^DN^ five were found to do so: Raspberry (Ras), Fatty acid binding protein (Fabp), PHGPx, Eukaryotic initiation factor 1A (EIF-1A) and Tan (T). We could only test Tan, as this was the only one of those five proteins for which antibodies were available [39], but when antibody was tested, and contrary to what would be expected from the MS data, this protein was found by Western blot to be less ubiquitinated when Rpn10^DN^ was overexpressed (panel E in S6 Fig). This result was not due to a loading error since anti-FK1 antibody immunoblot performed on the same membrane revealed that there is more polyubiquitinated material in the Rpn10^DN^ sample (panel C in S6 Fig). Despite great advances have been done in the MS field, the reliance on it as the unique technique to identify changes in ubiquitinated protein levels on different samples might still lead to obtaining false positives, and therefore emphasizes the importance of using orthogonal approaches along with MS.

### Validation of identified ubiquitination sites with a simple GFP-based pulldown protocol

**Fig 5:**
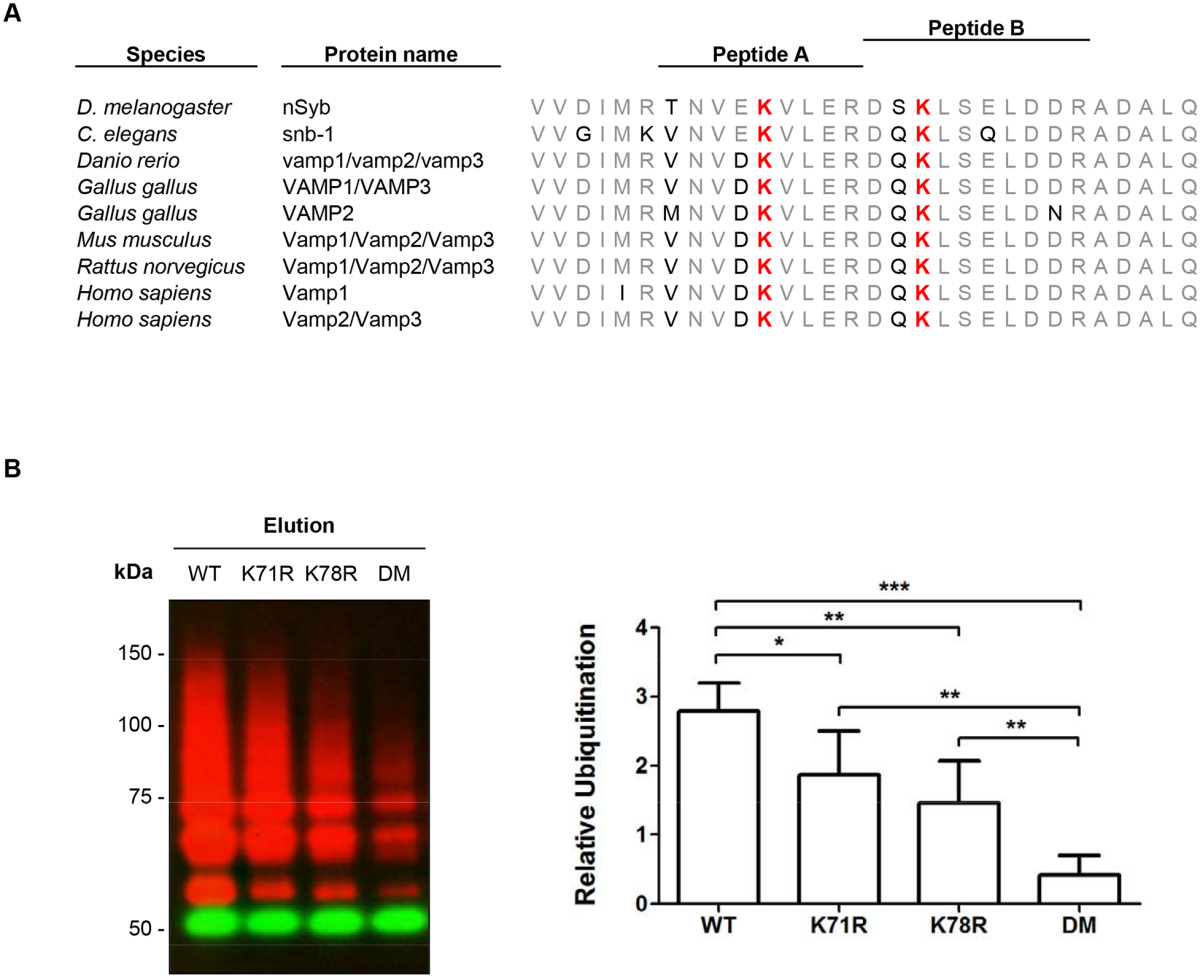
Ubiquitination sites of nSyb in neuronal cell line. (A) Ilustration of the peptides and the ubiquitination sites found in *Drosophila melanogaster* nSyb. The region of the protein where the modified lysines (red) were found is conserved among different species. The conserved aminoacids are shown in gray. (B) nSyb double mutant (DM) showed a significant reduction in its ubiquitinated fraction, as shown with anti-Flag antibody Western blot (red) compared to the wild type (WT) or the single lysine mutated forms (K71R or K78R). The non-modified form of nSyb was detected by GFP antibody (green). Quantification of the ubiquitination status of nSyb mutants relative to the non-modified form (Y axis: relative ubiquitination) was performed with Image-J. The plot shows relative levels of nSyb ubiquitination normalized to the GFP levels (average intensity ± SD). A t-test analysis for every pair of conditions was performed with GraphPad. One asterisk indicates p-value < to 0.05; two, p < to 0.01; three, p < to 0.0001.

The ^bio^Ub strategy does not particularly enrich for peptides carrying the di-gly signature that is left on ubiquitinated lysines after trypsin digestion. However, we did still find a total of 60 di-gly remnants (i.e. ubiquitination sites), in both embryonic and adult samples (Table 2). In order to confirm that these potential sites are indeed ubiquitinated, we applied a protocol that we have recently developed [27, 44, 58] to one of the identified proteins. We used neuronal cell culture to validate the ubiquitination of the neuronal synaptobrevin (nSyb) protein. The selection of nSyb for the validation of the ubiquitin sites identified was based on three criteria: first, the ubiquitination sites, K71 and K78, were detected twice in independent ^bio^Ub pulldowns. Second, those ubiquitination sites were found in a region of the protein that is conserved among *Drosophila* nSyb isoforms as well as with other organisms (Fig 5A), including humans [59]. And third, this protein has been widely studied because of its important role in neuronal proteins secretion [59]. The synaptic vesicle membrane protein nSyb belongs to a protein family known as SNARE (soluble N-ethylmaleimide-sensitive factor attachment protein receptor), which forms a complex with Syntaxin 1A and the Synaptosomal-associated protein 25 kDa (Snap25), two synaptic plasma membrane proteins also belonging the SNARE family (and found to be ubiquitnated in our list: see Fig 3B and S2 Table), that triggers membrane fusion and neuronal exocytosis [60]. Moreover, we used mutagenesis to confirm that the ubiquitination of this protein does indeed occur at those lysines. Interestingly, the lysines found to be ubiquitinated are located in a region of the protein that interacts with Syntaxin 1A and Snap25 (known as SNARE motif), specifically surrounding an arginine that is known to be essential for its function [59, 60].

**Table 2:** Ubiquitin modified peptides identified in the ^bio^Ub pulldown.

			PEP Score	
Protein	Peptide Sequence	Position of di-glycine	elav	GMR
Act42A	R ↓ VAPEEHPVLLTEAPLNPK (G-G) ANR ↓ E	K114	3.54 x 10^−003^	
Ben	R ↓ FITK (G-G) IYHPNIDR ↓ L	K74	5.82 x 10^−005^	
C11.1	R ↓ GK (G-G) VM*VPGAETIYAR ↓ C	K587		7.72 x 10^−003^
CG6652	R ↓ LEEK (G-G) DNDMK (G-G) LM*AR ↓ K	K210 K215	3.92 x 10^−002^	
CG7768	R ↓ SDVVPK (G-G) TAENFR ↓ A	K31	6.29 x 10^−002^	
CG8223	K ↓ GK (G-G) ELFSQGSR ↓ N	K60	5.21 x 10^−002^	
CG9899	M ↓ EK (G-G) MLSTYIEEAMEFYAIGK ↓ G	K3 (r)	7.97 x 10^−03^	
CG10550	R ↓ DLFESLGK (G-G) QR ↓ E	K214		3.14 x 10^−003^
CG12237	R ↓ K (G-G) GFAM*EK (G-G) HLLR ↓ N	K226(0.92) K232(0.08)	6.04 x 10^−002^	
CG13855	K ↓ EMGK (G-G) PIEWVGYK (G-G) DSK (G-G) ↓ I	K412(1) K420(0.975) K423(0.025)	4.70 x 10^−002^	
CG18538	K ↓ QTYFGNK (G-G) CVIDGDGLPEIVPAGFYLIVIK ↓ C	K38 (r)	1.67 x 10^−002^	
CG44252	R ↓ K (G-G) NLNIGDIFESNVAR ↓ R	K137		7.39 x 10^−011^
Df31	K ↓ VAAEEVDAVK (G-G) K (G-G) ↓ D	K26(0.858) K27(0.142)	1.77 x 10^−002^	
	K ↓ K (G-G) DAVAAEEVAAEK ↓ A	K27	7.03 x 10^−006^	
	R ↓ K (G-G) VDEAAAK (G-G) ADEAVATPEKK ↓ A	K142(0.03) K149(0.97)	3.87 x 10^−002^	
	K ↓ ADEAVATPEK (G-G) K (G-G) ↓ A	K159(0.978) K160(0.022)	3.59 x 10^−002^	
EIF-1A	R ↓ LEAMCFDGVK (G-G) R ↓ L	K56 (r)		9.67 x 10^−003^
	R ↓ DYQDSK (G-G) ADVILK ↓ Y	K88 (r)		8.25 x 10^−004^
	R ↓ NLK (G-G) TYGEFPESVR ↓ I	K104 (r)		3.17 x 10^−003^
Eps-15	K ↓ FQSK (G-G) EPVKDK ↓ F	K1221	1.37 x 10^−006^	
Fax	K ↓ SEAPPAQK (G-G) FNVHK ↓ T	K93		1.60 x 10^−006^
	K ↓ SEAPPAQK (G-G) FNVHK (G-G) ↓ T	K93(0.987) K98(0.013)		4.36 x 10^−003^
	K ↓ LDLNAHIPK (G-G) PEPETK ↓ E	K365	6.15 x 10^−004^	
	K ↓ SNEQEGTEGDK (G-G) IEK (G-G) ELEK (G-G) ↓ D	K392(0.008) K395(0.535) K399(0.457)	1.77 x 10^−125^	
His2A	K ↓ LLSGVTIAQGGVLPNIQAVLLPK (G-G) K (G-G) ↓ A	K118(0.5) K119(0.5)	6.43 x 10^−015^	
His2B	K ↓ AVTK (G-G) YTSSK	K118	2.13 x 10^−009^	
Hrb98DE	K ↓ LFVGALK (G-G) DDHDEQSIR ↓ D	K129	4.35 x 10^−005^	
Hsc70-1	R ↓ ITITNDK (G-G) GR ↓ L	K421		4.40 x 10^−005^
Hsc70-4	R ↓ IINEPTAAAIAYGLDK (G-G) K (G-G) ↓ A	K187(0.5) K188(0.5)	4.47 x 10^−002^	
	K ↓ ITITNDK (G-G) GR ↓ L	K507	1.38 x 10^−012^	
Hsp26	R ↓ IIQIQQVGPAHLNVK (G-G) ANESEVK (G-G) ↓ G	K189(0.988) K196(0.012)	4.76 x 10^−004^	
Nlp	K ↓ QILLGAEAK (G-G) ENEFNVVEVNTPK ↓ D	K44	5.53 x 10^−004^	
Nrv2	M ↓ (*ac*)SK (G-G) PVPM*SPSFVDEDLHNLR ↓ K	K3		8.69 x 10^−034^
nSyb	R ↓ TNVEK (G-G) VLER ↓ D	K71		8.94 x 10^−006^
	R ↓ DSK (G-G) LSELDDR ↓ A	K78		4.03 x 10^−007^
Pdh	K ↓ NAVVTGGAGGIGLQVSK (G-G) QLLAAGAAK(G-G) ↓ V	K40(0.993) K49(0.007)		5.42 x 10^−010^
	K ↓ K (G-G) GVEATYEEIAK ↓ T	K85		8.01 x 10^−009^
	R ↓ LNK (G-G) QSAADVSR ↓ C	K232		1.26 x 10^−003^
Rin	R ↓ NNK (G-G) GDFEQR ↓ R	K475	1.75 x 10^−003^	
RpS10b	R ↓ RAPGGSGVDK (G-G) K (G-G) GDVGPGAGEVEFR ↓ G	K137 K138	1.19 x 10^−007^	
RpS20	K ↓ DIEK (G-G) PHVGDSASVHR ↓ I	K10	3.21 x 10^−003^	
	M ↓ (*ac*)AAAPK (G-G) DIEK (G-G) PHVGDSASVHR ↓ I	K6 K10	3.21 x 10^−003^	
RpS27A	K ↓ VDENGK (G-G) IHR ↓ L	K113	2.85 x 10^−005^	
Sbb	K ↓ YK (G-G) HANGLR ↓ Y	K1124 (r)	2.11 x 10^−002^	
Scramb2	K ↓ LELLTGFETK (G-G) NR ↓ F	K82		5.09 x 10^−004^
	K ↓ VLSANNEEIGK (G-G) ISK ↓ Q	K214		7.38 x 10^−009^
Spn-F	R ↓ INIIQEK (G-G) IK ↓ A	K300		4.29 x 10^−003^
Syd	R ↓ ISELEDELK (G-G) K (G-G) AK ↓ E	K430 K431		1.34 x 10^−003^
Ubi/^bio^Ub	M*QIFVK (G-G) TLTGK ↓ T	K6 (r)		2.64 x 10^−022^
	K ↓ TLTGK (G-G) TITLEVEPSDTIENVK ↓ A	K11	1.03 x 10^−105^	1.82 x 10^−191^
	K ↓ TITLEVEPSDTIENVK (G-G) AK (G-G) ↓ I	K27(0.948) K29(0.052)	1.32 x 10^−035^	
	K ↓ TITLEVEPSDTIENVK (G-G) AK (G-G) ↓ I	K27(0.5) K29(0.5) (r)		1.02 x 10^−011^
	K ↓ AK (G-G) IQDKEGIPPDQQR ↓ L	K29	3.46 x 10^−302^	
	K ↓ IQDK (G-G) EGIPPDQQR ↓ L	K33		4.77 x 10^−006^
	R ↓ LIFAGK (G-G) QLEDGR ↓ T	K48	6.57 x 10^−176^	
	R ↓ LIFAGK (G-G) QLEDGRTLSDYNIQK ↓ E	K48		1.14 x 10^−044^
	R ↓ TLSDYNIQK (G-G) ESTLHLVLR ↓ L	K63	3.08 x 10^−068^	1.06 x 10^−032^
	R ↓ TLSDYNIQK (G-G) ESTLHLVLRLR ↓ G	K63	5.72 x 10^−111^	1.15 x 10^−300^

Ubiquitin modified peptides for 35 different proteins, including ubiquitin itself, were identified. Peptides sequences, positions of di-gly (G-G) with each probability in brackets (when different from 1) and Posterior Error Probabilities (PEP Score) are reported. Oxidized methionine is indicated by an asterisk (*) and acetylation by (*ac*). Some of those ubiquitination sites were only identified upon Rpn10^DN^ overexpression. Those ones are indicate by (r) in the position of di-glycine column. All peptides and intensities are shown in S1 and S2 Tables.

In order to validate the two ubiquitination sites of nSyb, *Drosophila* BG2 cells were transfected with either N- or C-terminally GFP-tagged nSyb and Flag-tagged ubiquitin. Cells were subjected to a GFP pulldown assay [41] and Western blot was performed with the eluted samples to confirm the expression and ubiquitination of GFP-tagged nSyb proteins (S9 Fig). The C-terminally GFP-tagged nSyb displayed a stronger ubiquitination as compared with the N-terminally GFP-tagged version and was, therefore, used for the generation of nSyb protein mutants carrying either the lysine 71 or the lysine 78 mutated to arginine (K71R and K78R) and a double mutant carrying both (DM). Ubiquitination of nSyb mutants was monitored by Western blot on GFP eluted nSyb from transfected cells. Both K71R and K78R mutants showed reduced ubiquitination, when compared to WT nSyb, as shown in Fig 5B (see also S9 Fig). Furthermore, there was a very significant reduction (p<0.0001) if both K were mutated at the same time, when compared to the wild-type levels. Despite homologous sites being earlier identified in a large scale MS analysis of rat brain [22], to our knowledge this is the first time that these ubiquitination sites have been biochemically validated. The role of ubiquitination on these lysines for nSyb function, however, still requires further characterization.

## Conclusions

The *Drosophila* eye has been widely used for the study of human neurodegenerative disorders because it provides a good system to perform unbiased genetic screens and analyse complex neural phenotypes, including behaviour [48]. At the same time it also offers the opportunity to study the functions carried out in mature neurons by mutating specific genes or pathways, without compromising the fertility and viability of the flies. In order to compare the differences in the ubiquitome between a developing and a differentiated neuron we therefore expanded our ^bio^Ub strategy [21] to the fly eye. This has allowed us to confirm that the ubiquitin proteome is not constant, and depends not just on the tissue studied, but also on the temporal context. While some overlap exists on the identified proteins, many others appear to be specific to the cell type (i.e. developing *vs* differentiated neuron), and more importantly, there are significant differences on the biological processes, cellular compartments and molecular functions targeted by the UPS. This reinforces the notion that observed differences are not a random occurrence, but describe instead the pathways that require to be regulated by ubiquitination at different periods during development and different cell types: e.g. in the embryonic neurons nuclear and cytoskeleton proteins are more represented, while in the adult neurons synaptic and membrane proteins are highly enriched. In parallel, proteins involved in transport and synaptic transmission are much more widespread in the adult than in the embryonic neurons, where cell cycle and developmental processes are more abundant. Similarly, transporter activity and nucleic acid binding functions are characteristic of the adult and embryo neuronal tissues, respectively. Ubiquitin proteomic studies tend to focus their efforts on increasing the amount of identified ubiquitinated proteins without paying much attention on the type of cell used. The need to improve the ubiquitome enrichment strategies clearly justify this; however, our results suggest that the use of the correct tissue or cell type is mandatory if particular pathways, such as those only found in neurons, are to be studied.

One powerful feature of our ^bio^Ub is that, unlike other approaches, it allows us to validate the ubiquitination status of the identified proteins by Western blot directly from the tissue of study. Using available specific antibodies we can determine whether a protein is being mono or polyubiquitinated, and further discriminate between those proteins modified through an amide bond with ubiquitin and those that are bound to it by a thioester linkage, which is the case for ubiquitin conjugating and ligating enzymes. Here we have validated the ubiquitination status of some of the proteins identified in the *Drosophila* eye (Fig 3B), as we did with some of the proteins identified during *Drosophila* nervous system development [21]. We have also confirmed that Parkin and Ube3a E3 ligases are ubiquitin carriers and remarkably, this tells us that these two proteins are not only expressed, but are active during the temporal window being studied.

Additionally, changes of ubiquitination based on different treatments can also be tested using the ^bio^Ub strategy, by immunoblotting for specific proteins in the purified samples. For example, here we combined the ^bio^Ub flies with a proteasomal shuttling factor mutant that acts as a dominant negative, which allowed us to compare the ubiquitinated material that is enriched. The next logical step will be to apply this strategy to models of disease, in order to identify specific proteins that are differentially ubiquitinated. The work presented here, including the observation that proteasomal inhibition can enrich the abundance of some ubiquitinated proteins, but reduce the abundance of others—particularly monoubiquitinated proteins-, will provide a baseline from which to perform such studies. Furthermore, the application of the GFP-pulldown based ubiquitination assay described here will allow for *in vivo* validation of the identified ubiquitination sites.

## Supporting Information

S1 Fig Expression of bioUb with various drivers to study adult brain ubiquitination. Anti-biotin Western blots were performed to monitor the expression of the ^UAS^(^bio^Ub)_6_-BirA construct in *Drosophila* adult heads using various GAL4 drivers. Flies were raised at 25°C, unless mentioned otherwise. (A) The pan-neuronal elav^GAL4^ (lane 1), the glutamatergic neuron-specific OK371^GAL4^ (lanes 2–4), the eye-specific GMR^GAL4^ (lane 5) and the temperature-sensitive Tub^GAL4,GAL80ts^ (lane 8) drivers were used to express ^bio^Ub, as well as a modified version of the ^bio^Ub construct (^UAS^GFP(^bio^Ub)_6_-BirA) in the case of OK371^GAL4^ (lane 4). Coexpression of ^UAS^Rpn10-ΔNTH (Rpn10^DN^) was tested with the elav^GAL4^ (lane 6) and OK371^GAL4^ (lanes 7) drivers in an attempt to accumulate more ubiquitinated material. Expression of ^bio^Ub with GMR^GAL4^ provided the highest expression. (B) Expression of the ^UAS^(^bio^Ub)_6_-birA construct was also tested using the Hs^GAL4^ driver and different heat shock treatments (lanes 3, 5–13). Among the different conditions tested, the highest expression was obtained when flies were born and kept at 29°C (lane 3). However, this condition did not reach the expression levels achieved with the GMR^GAL4^ driver at 25°C (lane 1). Six heads (three males and three females) were collected for each condition, but only a volume equivalent to 1 head was loaded per lane. Endogenous biotinylated *Drosophila* Acetyl-CoA carboxylase (ACC), Pyruvate carboxylase (CG1516) and biotin carboxylase (CG2118) are indicated with arrows.
(TIF)

S2 Fig Silver stainings of the material purified with the Neutravidin beads. Equal amounts of BirA and ^bio^Ub samples were analysed for each pulldown using SDS-PAGE, and stained with silver. Common bands between the two samples are expected to be composed mainly of endogenously biotinylated material, while the thick bands at around 40 kDa and below correspond to trimer, dimer and monomer forms of NeutrAvidin. The main high molecular weight smear observed in the experimental (^bio^Ub) but not in the control (BirA) samples corresponds to the isolated ubiquitinated material, more visibly seen among the four independent embryo replicates (A) than among the three replicates performed with the adult samples (B).
(TIF)

S3 Fig Anti-biotin Western blots used to monitor the purification process. Various dilutions of the input, flow-through (FT) and elution samples, as indicated, were loaded and monitored by Western blotting with anti-biotin both for embryo (A) and adult (B) in order to confirm the correct purification and enrichment of the ubiquitinated material as well as to estimate the recovery yield, which was in the range of 20–40% for all pulldowns. (C) Detection of ubiquitinated TBPH protein (arrow) from an adult pulldown. BirA: samples overexpressing the BirA enzyme; ^bio^Ub: samples overexpressing the construct carrying 6 copies of ubiquitin plus the BirA enzyme.
(TIF)

S4 Fig Venn diagrams indicate the overlap between the identified proteins in the several independent pulldown experiments performed. For each independent analysis every protein whose ^bio^Ub/BirA Label Free Quantification (LFQ) ratio was lower than four (LFQ intensity ^bio^Ub/BirA < 4) was considered background. In those situations where LFQ was not available, raw intensities were used to discriminate the background proteins, and the ^bio^Ub/BirA threshold ratio used was ten (raw intensity ^bio^Ub/BirA < 10) for proteins to be considered background. Proteins considered background in one biological replica but hit in another were only considered hits if the number of times classified as hit were higher than the times classified as background or if we had evidence of their ubiquitination. Each colour represents one independent pulldown experiment.
(TIF)

S5 Fig Box plots indicating the distribution of the identified proteins according to the maximum Label Free Quantification intensity recorded among different replica (LFQ). Box plots (A, B) show the distribution of the maximum LFQ intensities recorded (Y axis) and its positive correlation with the number of independent replica (X axis) on which those proteins appeared.
(TIF)

S6 Fig Effect of Rpn10^DN^ overexpression in the ubiquitinated material purified using the ^bio^Ub strategy. (A) Western blot analysis from embryo (elav^GAL4^) or adult heads (GMR^GAL4^) whole extract expressing the ^UAS^(^bio^Ub)_6_-birA construct alone (^bio^Ub) or together with Rpn10^DN^ (Rpn). Anti-biotin western blot clearly indicated an increase in the amount of the material that is ubiquitinated with the biotinylated ubiquitin when Rpn10^DN^ is expressed in embryos, as compared to expression of ^bio^Ub alone. In adults, a differential distribution is observed instead, with a preferential attachment of the biotinylated ubiquitin to higher molecular weight proteins. This effect is observed for similar expression levels of the ^UAS^(^bio^Ub)_6_-birA construct, as detected by anti-BirA antibody, indicating that the accumulation or the differential distribution of the ^bio^Ub conjugates is due to the overexpression of Rpn10^DN^. The expression of the Rpn10^DN^ construct was detected using an antibody to Rpn10 protein. (B) Anti-biotin Western blots with embryo (left) and adult (right) pulldown samples confirm that the same effect happens in the eluted fractions upon Rpn10^DN^ expression. Dilutions of the input, flow through (FT) and elution are shown. (C) An anti-FK1 immunoblot with the material eluted from adult heads indicated that the differential distribution observed with anti-biotin is also accompanied by an increased in the polyubiquitin chains when Rpn10^DN^ is expressed. The anti-FK1 western blot was performed on the same membrane used for the detection of ubiquitinated tan protein (see S6E). (D) Western blot to some known monoubiquitinated proteins confirmed that in Rpn10^DN^ samples the biotinylated ubiquitin is preferentially attached to polyubiquitinated proteins, in both embryo and adult samples. Analyses of monoubiquitinated Liquid facets (Lfq) and Neurotactin (Nrt) were performed in embryo pulldowns, while for monoubiquitinated Na pump alpha subunit (Atpα) and Failed axon connections (Fax) adult samples were used. (E) Western blot to Tan protein with adult ^bio^Ub and Rpn10^DN^ samples. BirA: flies expressing only the BirA enzyme; ^bio^Ub: flies expressing the ^UAS^(^bio^Ub)_6_-BirA construct; Rpn: flies expressing both the ^UAS^(^bio^Ub)_6_-BirA construct and the C-terminal half of Rpn10 protein (Rpn10^DN^).
(TIF)

S7 Fig Silver staining of the material purified with the Neutravidin beads for Rpn10^DN^ and ^bio^Ub only samples. Equal amounts of ^bio^Ub and ^bio^Ub+Rpn10^DN^ samples were analysed for each pulldown using SDS-PAGE and stained with silver. Both for embryo (A) and adult (B) samples an accumulation of ubiquitinated material is detected on samples from flies overexpressing the C-terminal half of Rpn10 (Rpn10^DN^) compared to the ^bio^Ub flies.
(TIF)

S8 Fig Venn diagrams indicate the overlap of proteins identified in ^bio^Ub and Rpn10^DN^ samples. Total amount of proteins identified by mass spectrometry from the ubiquitinated material isolated from embryo and adult Rpn10^DN^ samples was in both cases lower than the amount of proteins identified in ^bio^Ub samples.
(TIF)

S9 Fig Neuronal Synaptobrevin GFP-pulldown. Anti-Flag (red) and anti-GFP (green) Western blots performed with (A) C-terminally (C) or N-terminally (N) GFP-tagged WT nSyb. (B) With input samples from the GFP pulldown performed in Fig 5. And (C) with inputs and elutions of different independent GFP pulldowns carried out with C-terminal GFP-tagged nSyb mutants. WT: C-terminal GFP-tagged WT nSyb; K71R: C-terminal GFP-tagged nSyb where lysine 71 (K71) has been mutated to arginine (R); K78R: C-terminal GFP-tagged nSyb where lysine 78 (K78) has been mutated to arginine (R); DM: C-terminal GFP-tagged nSyb where both lysines (K71 and K78) have been mutated to arginine (R).
(TIF)

S1 Table Proteins identified by MS in embryo samples. Peptides, intensities and LFQ intensities of the proteins identified by MS among the different pulldown experiments are provided. Proteins classified as background or as ubiquitin conjugate are provided in separate sheet. A summary of the number of times each protein has been identified (based on peptides) in independent analysis for BirA (n BirA), ^bio^Ub (n bioUb) and Rpn10^DN^ (n Rpn10) is provided. Ubiquitination sites are shown in a separate sheet. Only ubiquitination sites for which intensity was recorded were taken as valid.
(XLS)

S2 Table Proteins identified by MS in adult samples. Peptides, intensities and LFQ intensities of the proteins identified by MS among the different pulldown experiments are provided. Proteins classified as background or as ubiquitin conjugate are provided in separate sheet. A summary of the number of times each protein has been identified (based on peptides) in independent analysis for BirA (n BirA), ^bio^Ub (n bioUb) and Rpn10^DN^ (n Rpn10) is provided. Ubiquitination sites are shown in a separate sheet. Only ubiquitination sites for which intensity was recorded were taken as valid.
(XLS)

S3 Table G:profiler Analysis. Proteins found only in embryo or only in adult were analysed by G:profiler for GO Term enrichment analysis. Summary of the enriched GO Terms in the Biological Process (BP), Cellular Compartment (CC) and Molecular Function (MF) domains are shown. Statistical enrichment of each Term is provided by the p-value, which is also represented by a colour according to its value (the lower the p-value the stronger intensity of red). The software calculates p-values using Fisher´s one tailed test combined with a custom multiple testing correction algorithm [46].
(PDF)
