# Supplementary figures and images for: Proteomic Analysis of the Ubiquitin Landscape in the Drosophila Embryonic Nervous System and the Adult Photoreceptor Cells
Source: PLoS One. 2015 Oct 13;10(10):e0139083. doi: 10.1371/journal.pone.0139083 (PMC4604154; doi:10.1371/journal.pone.0139083)

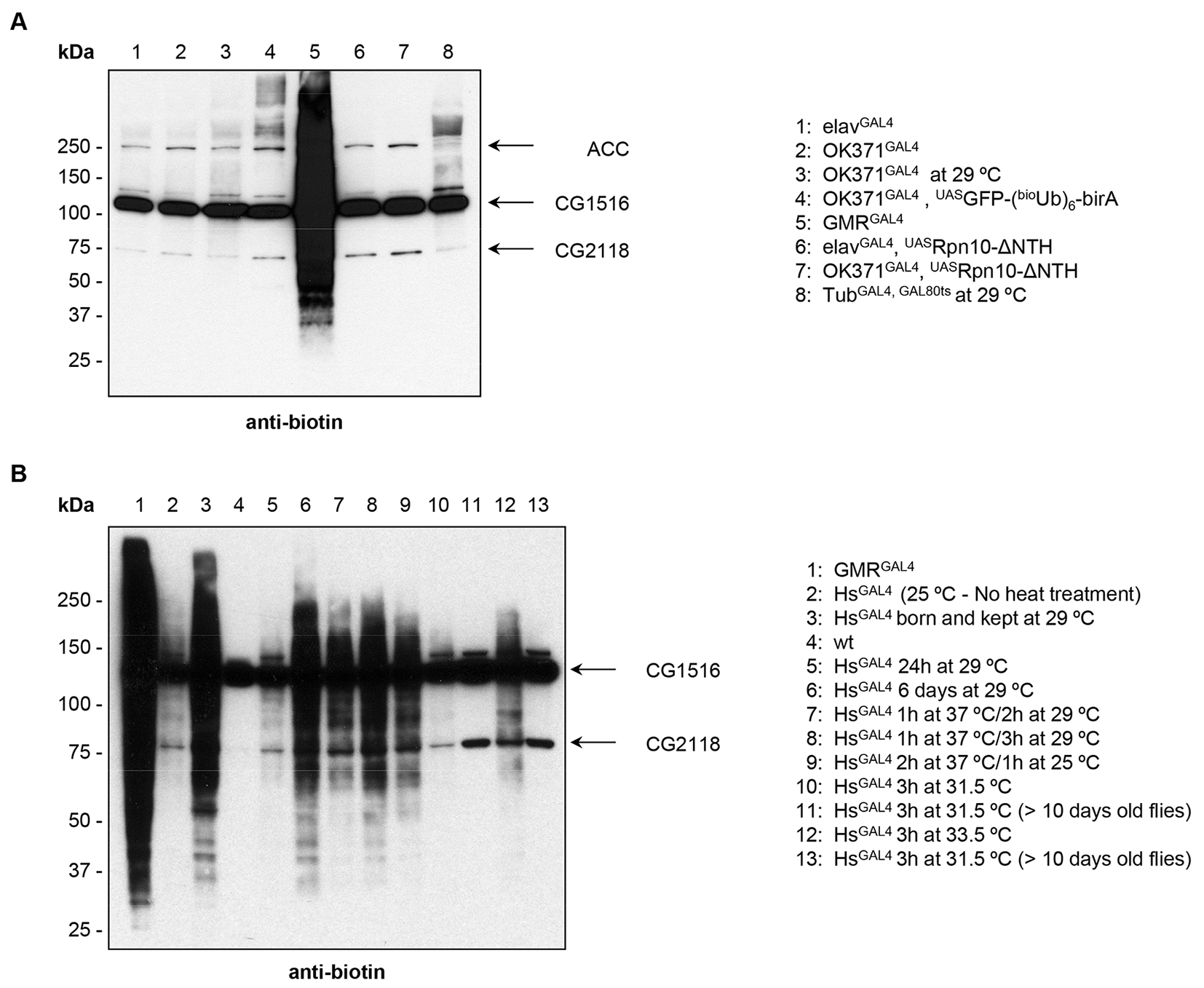

Supplement: S1 Fig — Anti-biotin Western blots were performed to monitor the expression of the UAS(bioUb)6-BirA construct in Drosophila adult heads using various GAL4 drivers. Flies were raised at 25°C, unless mentioned otherwise. (A) The pan-neuronal elavGAL4 (lane 1), the glutamatergic neuron-specific OK371GAL4 (lanes 2–4), the eye-specific GMRGAL4 (lane 5) and the temperature-sensitive TubGAL4,GAL80ts (lane 8) drivers were used to express bioUb, as well as a modified version of the bioUb construct (UASGFP(bioUb)6-BirA) in the case of OK371GAL4 (lane 4). Coexpression of UASRpn10-ΔNTH (Rpn10DN) was tested with the elavGAL4 (lane 6) and OK371GAL4 (lanes 7) drivers in an attempt to accumulate more ubiquitinated material. Expression of bioUb with GMRGAL4 provided the highest expression. (B) Expression of the UAS(bioUb)6-birA construct was also tested using the HsGAL4 driver and different heat shock treatments (lanes 3, 5–13). Among the different conditions tested, the highest expression was obtained when flies were born and kept at 29°C (lane 3). However, this condition did not reach the expression levels achieved with the GMRGAL4 driver at 25°C (lane 1). Six heads (three males and three females) were collected for each condition, but only a volume equivalent to 1 head was loaded per lane. Endogenous biotinylated Drosophila Acetyl-CoA carboxylase (ACC), Pyruvate carboxylase (CG1516) and biotin carboxylase (CG2118) are indicated with arrows. (TIF) [file pone.0139083.s001.tif]

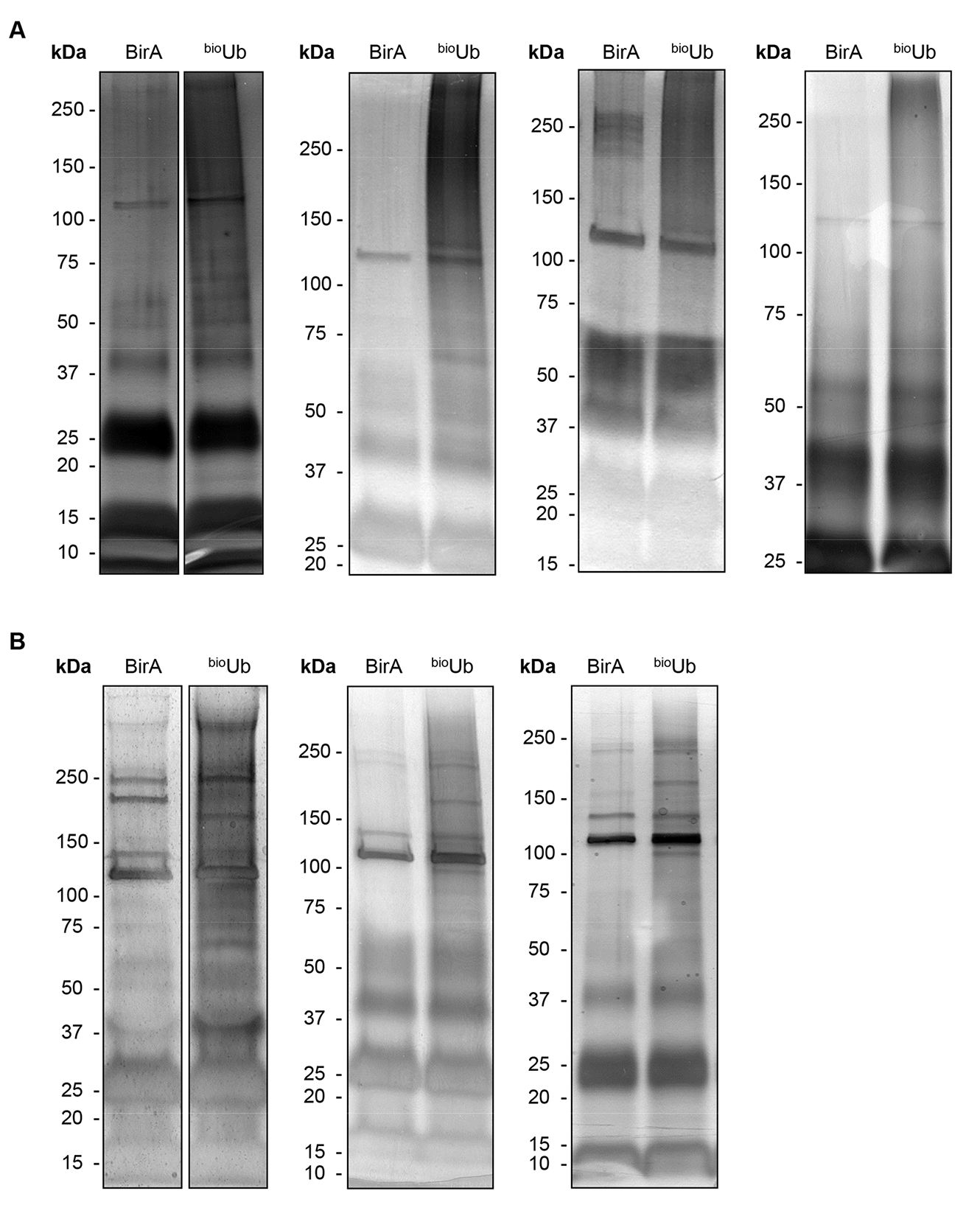

Supplement: S2 Fig — Equal amounts of BirA and bioUb samples were analysed for each pulldown using SDS-PAGE, and stained with silver. Common bands between the two samples are expected to be composed mainly of endogenously biotinylated material, while the thick bands at around 40 kDa and below correspond to trimer, dimer and monomer forms of NeutrAvidin. The main high molecular weight smear observed in the experimental (bioUb) but not in the control (BirA) samples corresponds to the isolated ubiquitinated material, more visibly seen among the four independent embryo replicates (A) than among the three replicates performed with the adult samples (B). (TIF) [file pone.0139083.s002.tif]

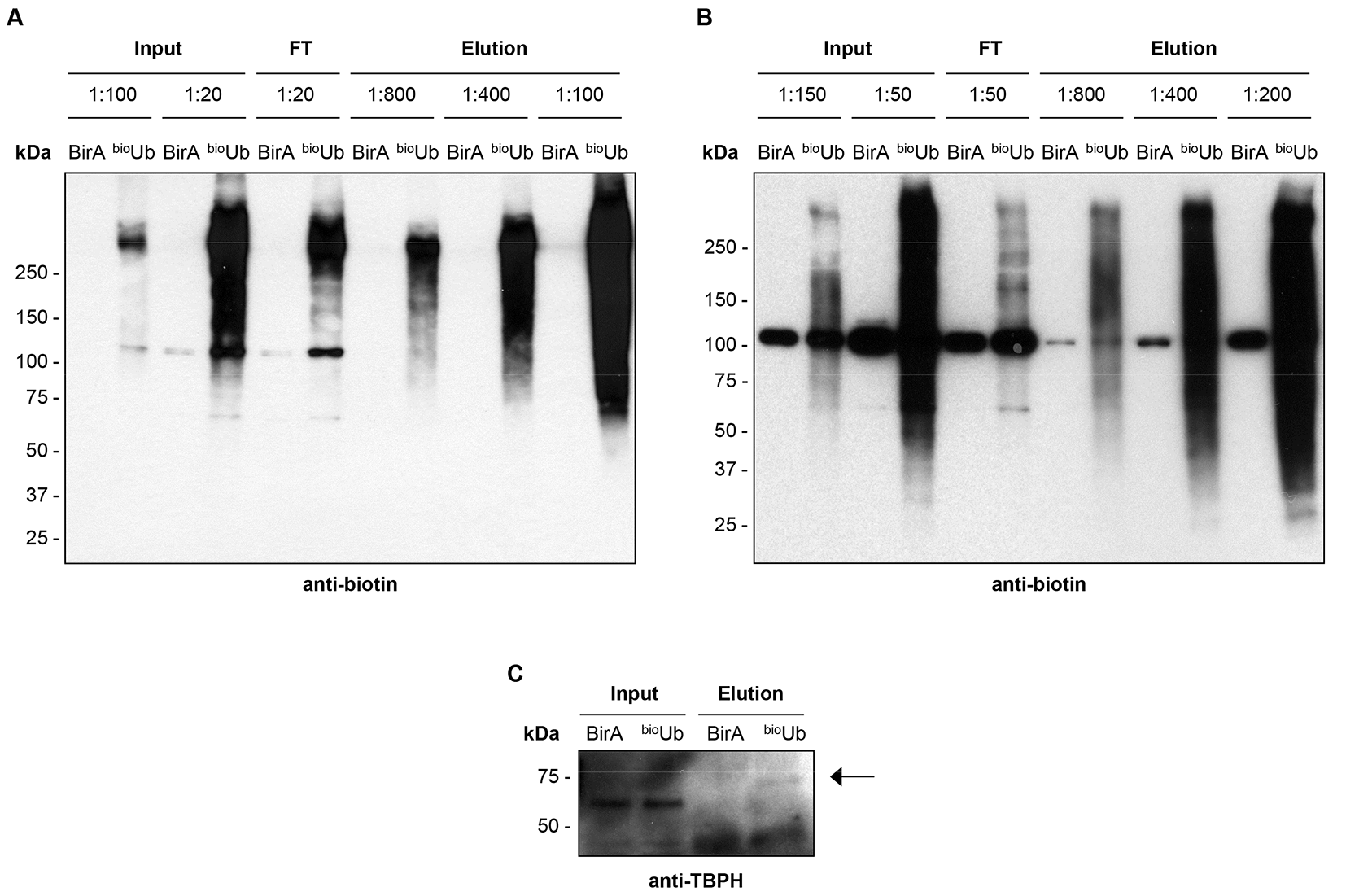

Supplement: S3 Fig — Various dilutions of the input, flow-through (FT) and elution samples, as indicated, were loaded and monitored by Western blotting with anti-biotin both for embryo (A) and adult (B) in order to confirm the correct purification and enrichment of the ubiquitinated material as well as to estimate the recovery yield, which was in the range of 20–40% for all pulldowns. (C) Detection of ubiquitinated TBPH protein (arrow) from an adult pulldown. BirA: samples overexpressing the BirA enzyme; bioUb: samples overexpressing the construct carrying 6 copies of ubiquitin plus the BirA enzyme. (TIF) [file pone.0139083.s003.tif]

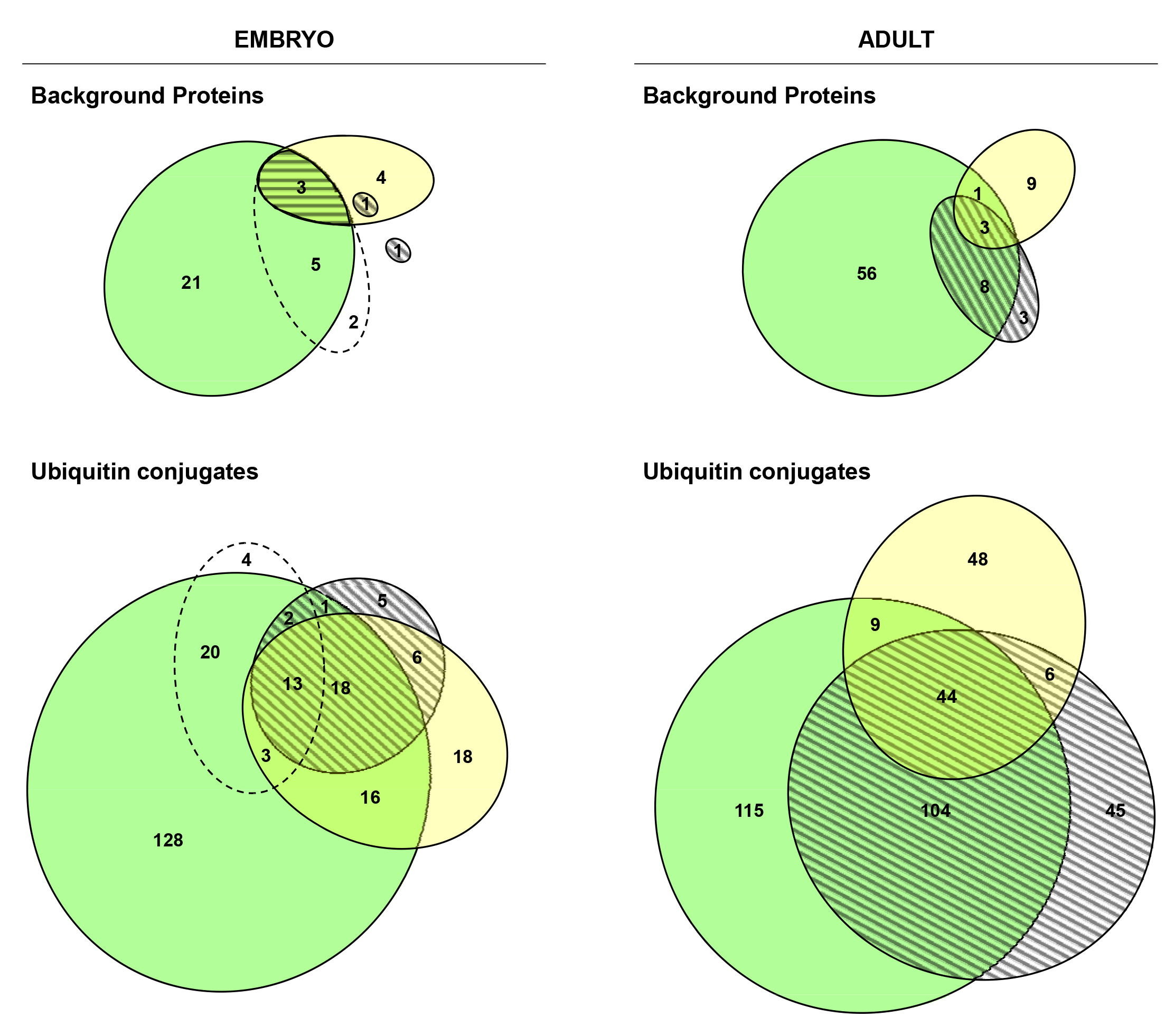

Supplement: S4 Fig — For each independent analysis every protein whose bioUb/BirA Label Free Quantification (LFQ) ratio was lower than four (LFQ intensity bioUb/BirA < 4) was considered background. In those situations where LFQ was not available, raw intensities were used to discriminate the background proteins, and the bioUb/BirA threshold ratio used was ten (raw intensity bioUb/BirA < 10) for proteins to be considered background. Proteins considered background in one biological replica but hit in another were only considered hits if the number of times classified as hit were higher than the times classified as background or if we had evidence of their ubiquitination. Each colour represents one independent pulldown experiment. (TIF) [file pone.0139083.s004.tif]

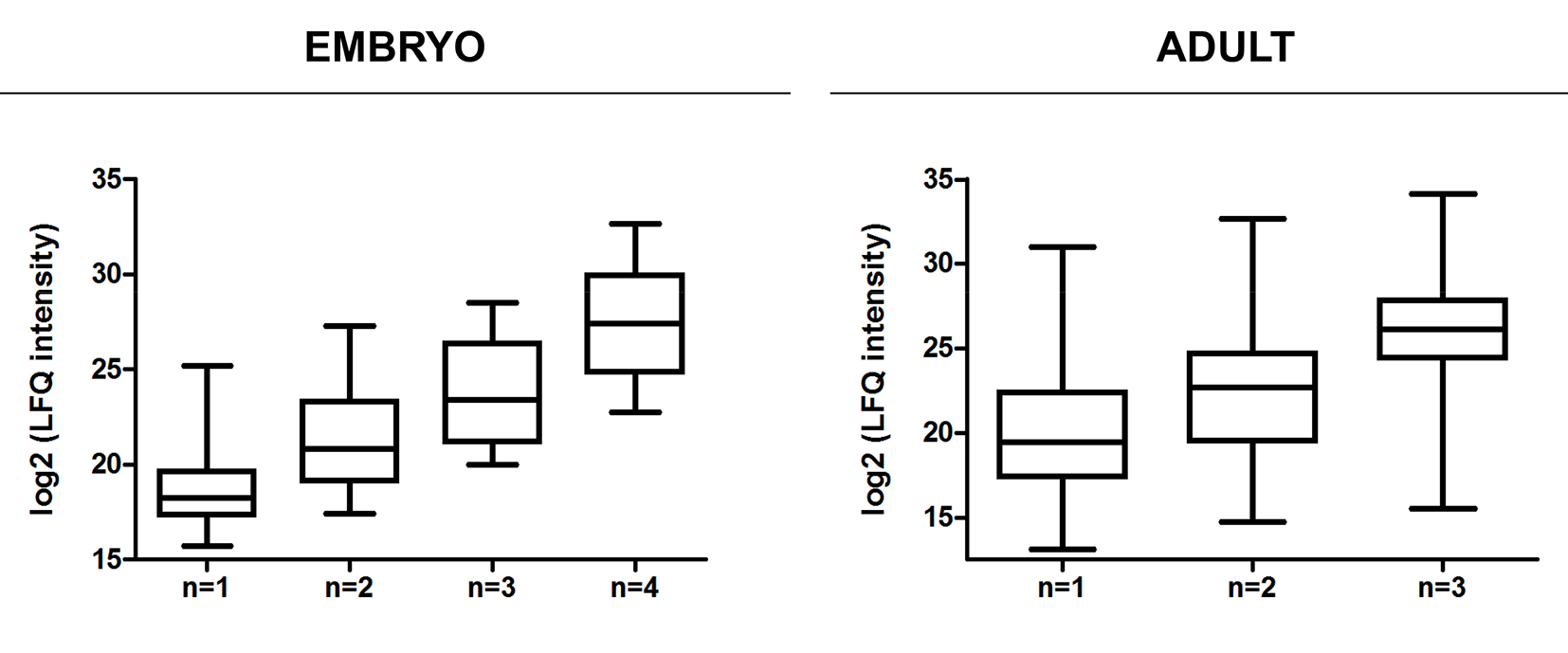

Supplement: S5 Fig — Box plots (A, B) show the distribution of the maximum LFQ intensities recorded (Y axis) and its positive correlation with the number of independent replica (X axis) on which those proteins appeared. (TIF) [file pone.0139083.s005.tif]

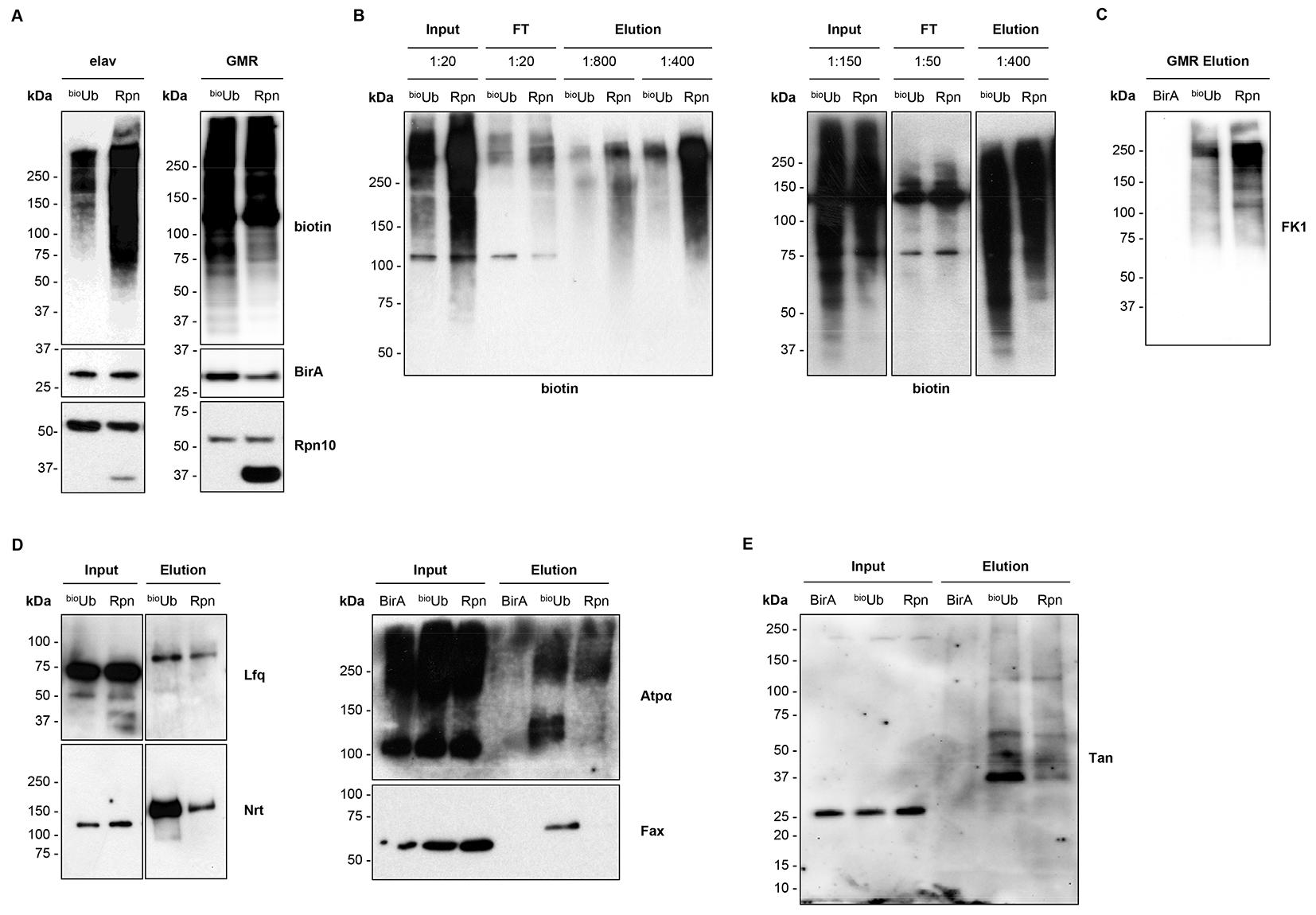

Supplement: S6 Fig — (A) Western blot analysis from embryo (elavGAL4) or adult heads (GMRGAL4) whole extract expressing the UAS(bioUb)6-birA construct alone (bioUb) or together with Rpn10DN (Rpn). Anti-biotin western blot clearly indicated an increase in the amount of the material that is ubiquitinated with the biotinylated ubiquitin when Rpn10DN is expressed in embryos, as compared to expression of bioUb alone. In adults, a differential distribution is observed instead, with a preferential attachment of the biotinylated ubiquitin to higher molecular weight proteins. This effect is observed for similar expression levels of the UAS(bioUb)6-birA construct, as detected by anti-BirA antibody, indicating that the accumulation or the differential distribution of the bioUb conjugates is due to the overexpression of Rpn10DN. The expression of the Rpn10DN construct was detected using an antibody to Rpn10 protein. (B) Anti-biotin Western blots with embryo (left) and adult (right) pulldown samples confirm that the same effect happens in the eluted fractions upon Rpn10DN expression. Dilutions of the input, flow through (FT) and elution are shown. (C) An anti-FK1 immunoblot with the material eluted from adult heads indicated that the differential distribution observed with anti-biotin is also accompanied by an increased in the polyubiquitin chains when Rpn10DN is expressed. The anti-FK1 western blot was performed on the same membrane used for the detection of ubiquitinated tan protein (see S6E). (D) Western blot to some known monoubiquitinated proteins confirmed that in Rpn10DN samples the biotinylated ubiquitin is preferentially attached to polyubiquitinated proteins, in both embryo and adult samples. Analyses of monoubiquitinated Liquid facets (Lfq) and Neurotactin (Nrt) were performed in embryo pulldowns, while for monoubiquitinated Na pump alpha subunit (Atpα) and Failed axon connections (Fax) adult samples were used. (E) Western blot to Tan protein with adult bioUb and Rpn10DN samples. BirA: fl [file pone.0139083.s006.tif]

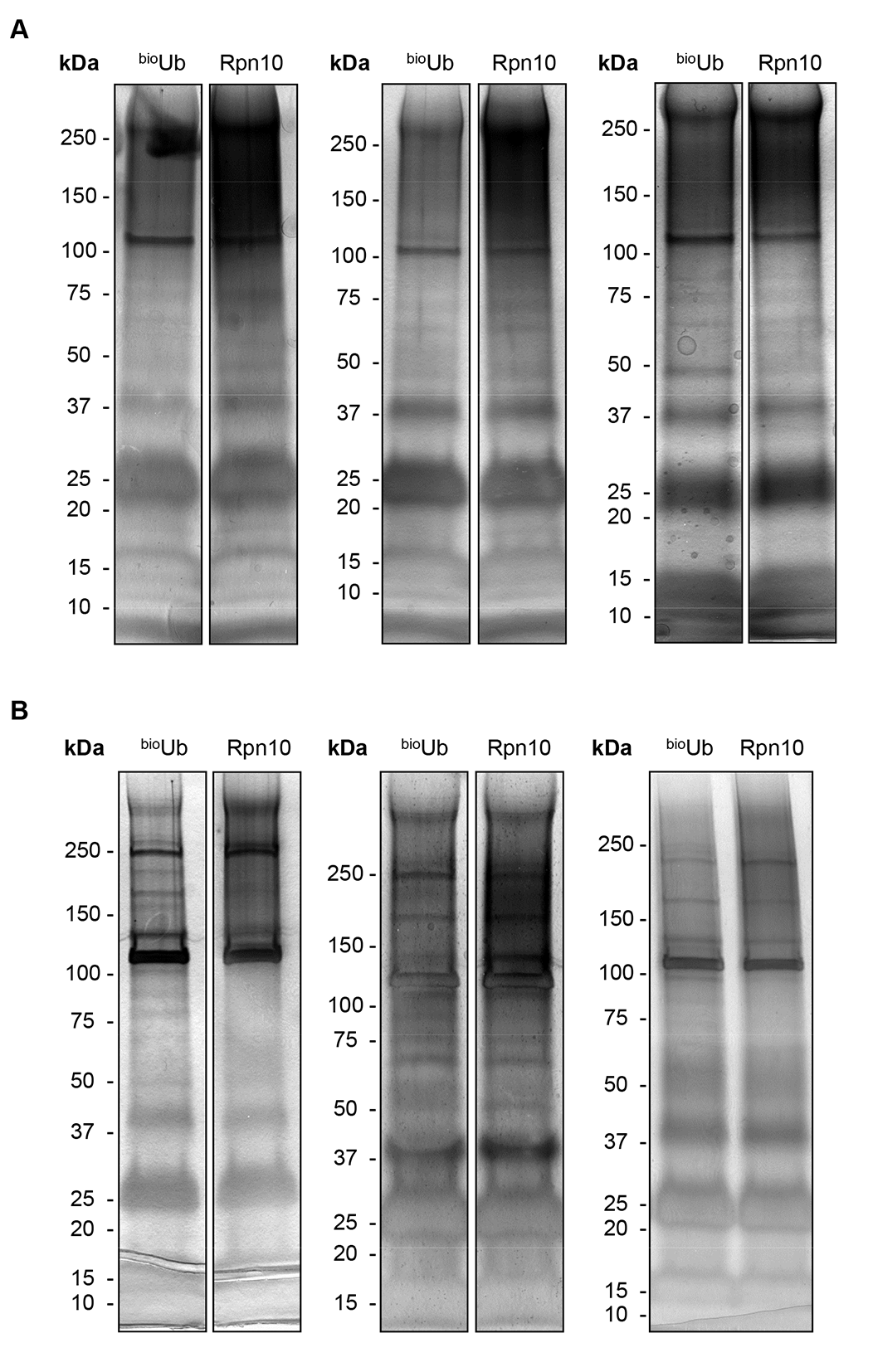

Supplement: S7 Fig — Equal amounts of bioUb and bioUb+Rpn10DN samples were analysed for each pulldown using SDS-PAGE and stained with silver. Both for embryo (A) and adult (B) samples an accumulation of ubiquitinated material is detected on samples from flies overexpressing the C-terminal half of Rpn10 (Rpn10DN) compared to the bioUb flies. (TIF) [file pone.0139083.s007.tif]

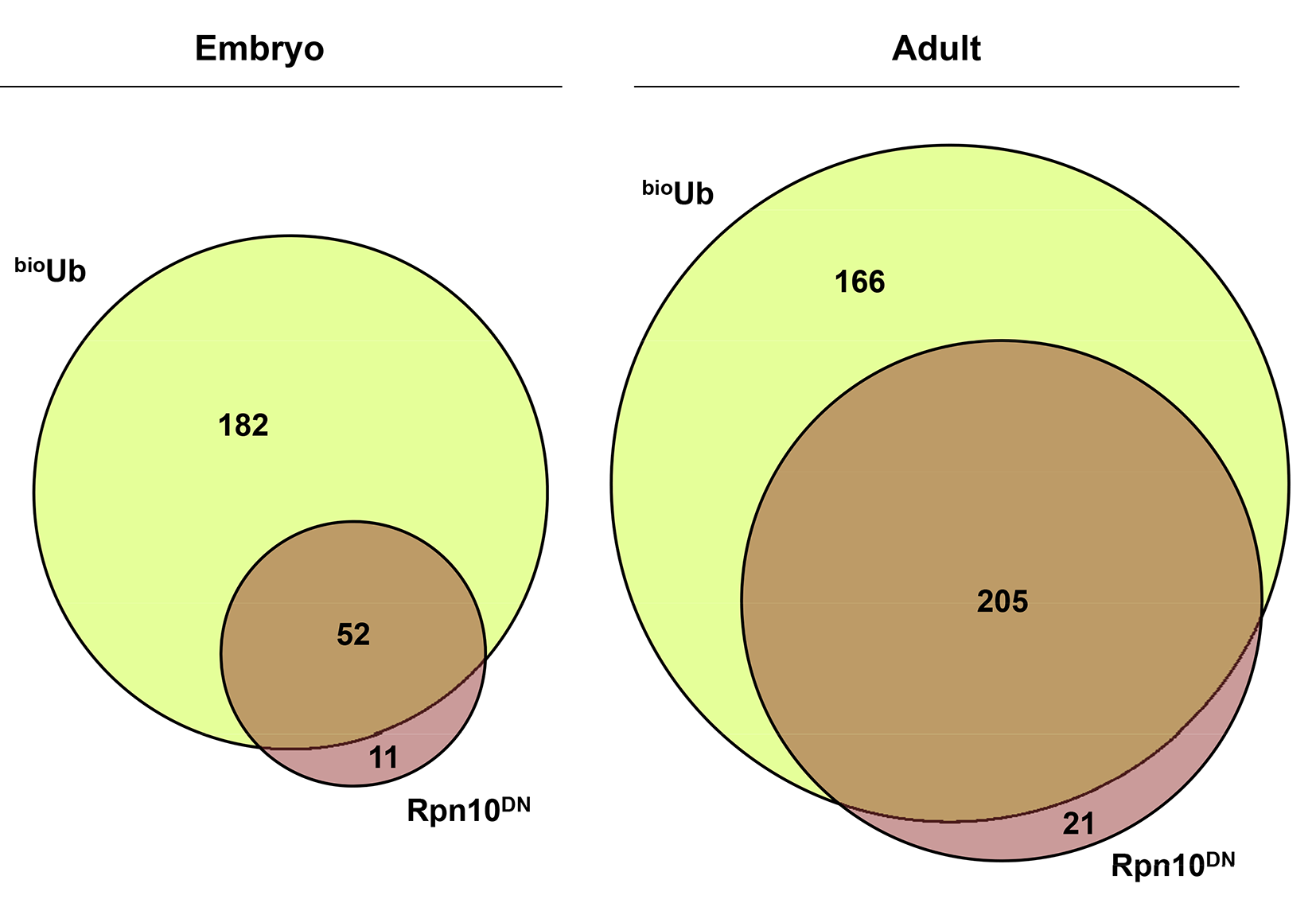

Supplement: S8 Fig — Total amount of proteins identified by mass spectrometry from the ubiquitinated material isolated from embryo and adult Rpn10DN samples was in both cases lower than the amount of proteins identified in bioUb samples. (TIF) [file pone.0139083.s008.tif]

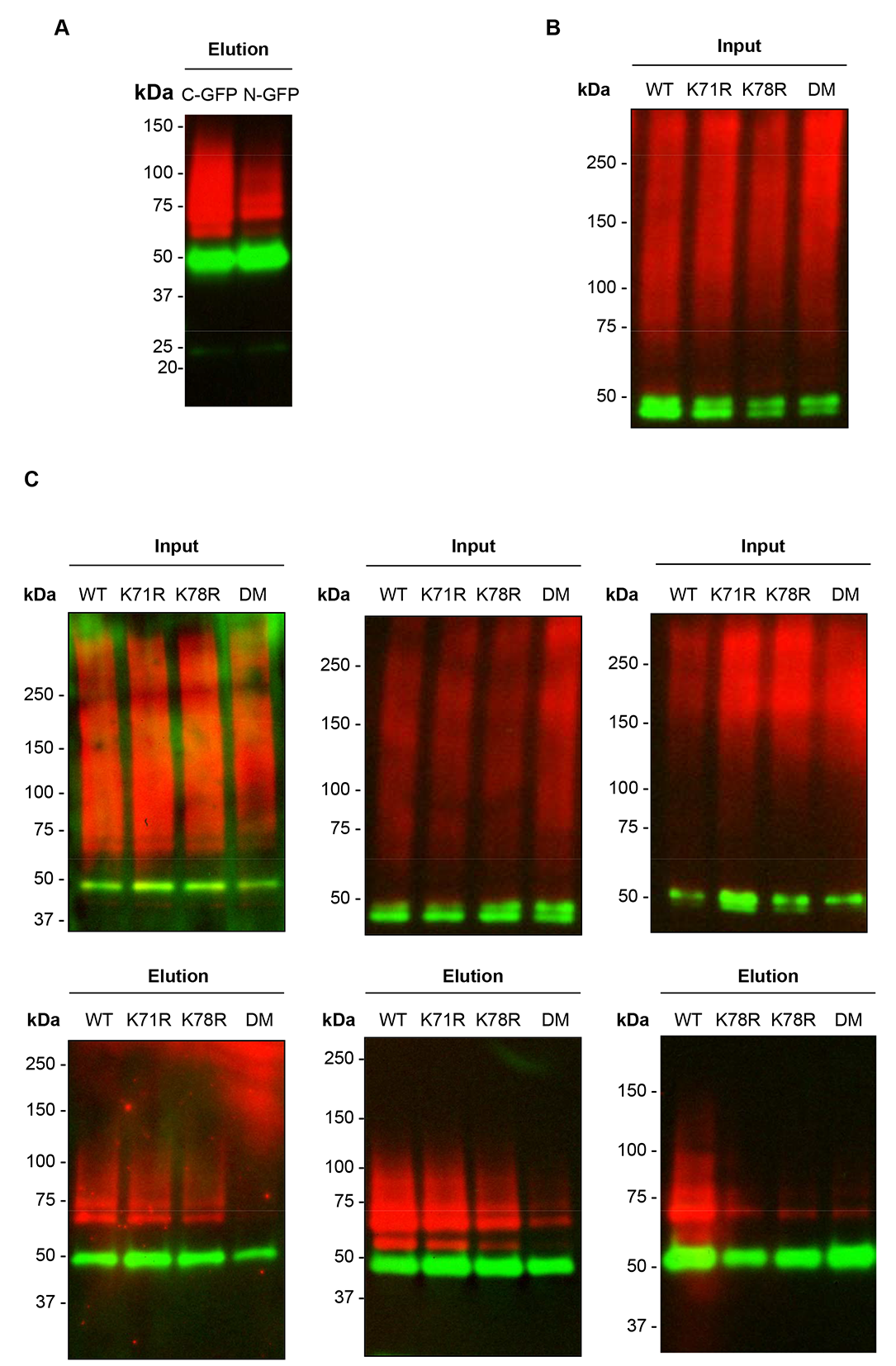

Supplement: S9 Fig — Anti-Flag (red) and anti-GFP (green) Western blots performed with (A) C-terminally (C) or N-terminally (N) GFP-tagged WT nSyb. (B) With input samples from the GFP pulldown performed in Fig 5. And (C) with inputs and elutions of different independent GFP pulldowns carried out with C-terminal GFP-tagged nSyb mutants. WT: C-terminal GFP-tagged WT nSyb; K71R: C-terminal GFP-tagged nSyb where lysine 71 (K71) has been mutated to arginine (R); K78R: C-terminal GFP-tagged nSyb where lysine 78 (K78) has been mutated to arginine (R); DM: C-terminal GFP-tagged nSyb where both lysines (K71 and K78) have been mutated to arginine (R). (TIF) [file pone.0139083.s009.tif]
