# Supplementary material for: Proteomic Analysis of the Ubiquitin Landscape in the Drosophila Embryonic Nervous System and the Adult Photoreceptor Cells
Source: PLoS One. 2015 Oct 13;10(10):e0139083. doi: 10.1371/journal.pone.0139083 (PMC4604154; doi:10.1371/journal.pone.0139083)
Supplement: S3 Table — Proteins found only in embryo or only in adult were analysed by G:profiler for GO Term enrichment analysis. Summary of the enriched GO Terms in the Biological Process (BP), Cellular Compartment (CC) and Molecular Function (MF) domains are shown. Statistical enrichment of each Term is provided by the p-value, which is also represented by a colour according to its value (the lower the p-value the stronger intensity of red). The software calculates p-values using Fisher´s one tailed test combined with a custom multiple testing correction algorithm [46]. (PDF) [file pone.0139083.s012.pdf]

Embryo p-value	Adult p-value	Go term	Go Term description	
3,84E-06	2,12E-02	GO:0009987	**cellular process**	BP
2,68E-07	1,00E+00	GO:0007049	cell cycle	BP
1,49E-03	1,00E+00	GO:0044237	cellular metabolic process	BP
4,58E-07	1,00E+00	GO:0006725	cellular aromatic compound metabolic process	BP
5,31E-06	1,00E+00	GO:0034641	cellular nitrogen compound metabolic process	BP
4,21E-07	1,00E+00	GO:0006139	nucleobase-containing compound metabolic process	BP
9,13E-08	1,05E-01	GO:0032502	**developmental process**	BP
1,75E-07	7,57E-01	GO:0048856	anatomical structure development	BP
7,78E-09	1,13E-01	GO:0007275	multicellular organismal development	BP
2,24E-10	4,17E-01	GO:0071840	**cellular component organization or biogenesis**	BP
1,91E-07	1,00E+00	GO:0044085	cellular component biogenesis	BP
6,01E-11	1,81E-01	GO:0016043	cellular component organization	BP
5,64E-12	4,56E-01	GO:0071841	cellular component organization or biogenesis at cellular level	BP
1,66E-12	2,25E-01	GO:0071842	cellular component organization at cellular level	BP
1,00E+00	1,49E-15	GO:0051179	**localization**	BP
1,43E-06	5,10E-03	GO:0032501	**multicellular organismal process**	BP
1,98E-07	6,57E-04	GO:0044707	single-multicellular organism process	BP
1,00E+00	6,40E-09	GO:0035637	multicellular organismal signaling	BP
1,00E+00	6,40E-09	GO:0019226	transmission of nerve impulse	BP
1,00E+00	3,45E-09	GO:0007268	synaptic transmission	BP
3,65E-03	1,45E-04	GO:0065007	**biological regulation**	BP
1,00E+00	7,81E-13	GO:0065008	regulation of biological quality	BP
1,00E+00	5,18E-13	GO:0051234	**establishment of localization**	BP
1,00E+00	2,53E-12	GO:0006810	transport	BP
4,37E-12	1,76E-01	GO:0005623	**cell**	CC
7,93E-16	1,00E+00	GO:0032991	**macromolecular complex**	CC
3,06E-11	1,00E+00	GO:0043226	**organelle**	CC
3,61E-17	1,00E+00	GO:0044422	**organelle part**	CC
4,37E-12	1,76E-01	GO:0044464	**cell part**	CC
5,08E-12	1,00E+00	GO:0005622	intracellular	CC
1,90E-13	1,00E+00	GO:0044424	intracellular part	CC
2,72E-11	1,00E+00	GO:0043229	intracellular organelle	CC
1,17E-14	1,00E+00	GO:0043232	intracellular non-membrane-bounded organelle	CC
1,05E-15	9,76E-01	GO:0005856	cytoskeleton	CC
1,81E-02	1,00E+00	GO:0043231	intracellular membrane-bounded organelle	CC
2,45E-07	1,00E+00	GO:0005634	nucleus	CC
6,04E-16	1,00E+00	GO:0044446	intracellular organelle part	CC
1,56E-06	1,00E+00	GO:0044428	nuclear part	CC
5,33E-16	1,00E+00	GO:0044430	cytoskeletal part	CC
1,00E+00	4,85E-16	GO:0071944	cell periphery	CC
1,00E+00	7,17E-18	GO:0005886	plasma membrane	CC
1,00E+00	6,97E-05	GO:0045202	**synapse**	CC
1,00E+00	1,03E-08	GO:0016020	**membrane**	CC
1,00E+00	6,27E-04	GO:0005215	**transporter activity**	MF
1,00E+00	3,43E-02	GO:0022857	transmembrane transporter activity	MF
1,00E+00	1,94E-03	GO:0022804	active transmembrane transporter activity	MF
1,00E+00	7,02E-04	GO:0015399	primary active transmembrane transporter activity	MF
1,00E+00	7,02E-04	GO:0015405	P-P-bond-hydrolysis-driven transmembrane transporter activity	MF
1,00E+00	5,21E-04	GO:0016820	hydrolase activity, catalyzing transmembrane movement of substances	MF
1,00E+00	5,21E-04	GO:0042626	ATPase activity, coupled to transmembrane movement of substances	MF
6,96E-02	1,00E+00	GO:0005488	**binding**	MF
6,45E-05	7,00E-02	GO:0005515	protein binding	MF
	1,21E-04	GO:0005484	SNAP receptor activity	MF
7,62E-03	1,00E+00	GO:1901363	heterocyclic compound binding	MF
5,16E-05	1,00E+00	GO:0003676	nucleic acid binding	MF
3,16E-03	1,00E+00	GO:0003723	RNA binding	MF
8,29E-04	1,00E+00	GO:0003729	mRNA binding	MF
